# Bile Acids Are Potential Negative Allosteric Modulators of M1 Muscarinic Receptors

**DOI:** 10.3390/biom15091326

**Published:** 2025-09-17

**Authors:** Wenbo Yu, Alexander D. MacKerell, David J. Weber, Jean-Pierre Raufman

**Affiliations:** 1 Computer-Aided Drug Design Center, Department of Pharmaceutical Sciences, School of Pharmacy, University of Maryland Baltimore, Baltimore, MD 21201, USA; amackerell@rx.umaryland.edu; 2 Institute for Bioscience and Biotechnology Research (IBBR), Rockville, MD 20850, USA; dweber@som.umaryland.edu; 3 Center for Biomolecular Therapeutics (CBT), School of Medicine, University of Maryland Baltimore, Baltimore, MD 21201, USA; 4 Department of Biochemistry and Molecular Biology, University of Maryland School of Medicine, Baltimore, MD 21201, USA; 5 Marlene and Stewart Greenebaum Cancer Center, University of Maryland School of Medicine, Baltimore, MD 21201, USA; 6 VA Maryland Healthcare System, Baltimore, MD 21201, USA; 7 Department of Medicine, Division of Gastroenterology and Hepatology, University of Maryland School of Medicine, Baltimore, MD 21201, USA

**Keywords:** bile acids, muscarinic receptors, cholinergic signaling, allosteric binding, site identification by ligand competitive saturation, docking, molecular dynamics

## Abstract

The proposed physiological roles of bile acids have expanded beyond the digestion of fats to encompass cell signaling via the activation of a variety of nuclear and plasma membrane receptors in multiple organ systems. The current in silico study was inspired by previous observations from our group and others that bile acids interact functionally with cardiac, pulmonary, and gastrointestinal muscarinic receptors and more recent work demonstrating allosteric binding of cholesterol, the parent molecule for bile acid synthesis, to M_1_ muscarinic receptors (M_1_R). Here, we computationally tested the hypothesis that bile acids can allosterically bind to M_1_R and thereby modulate receptor activation. Utilizing de novo site identification by the ligand competitive saturation (SILCS) method, putative novel allosteric binding sites of bile acid targeting M_1_R were identified. Molecular dynamics simulations were used to uncover the molecular details of the activation mechanism of M_1_R due to agonist binding along with allosteric modulation of bile acids on M_1_R activation. Allosteric binding of bile acids and their glycine and taurine conjugates to M_1_R negatively impacts the activation process, findings consistent with recent reports that M_1_R expression and activation inhibit colon cancer cell proliferation. Thus, bile acids may augment colon cancer risk by inhibiting the tumor suppressor actions of M_1_R. When validated experimentally, these findings are anticipated to shed light on our understanding of how bile acids in the membrane microenvironment can allosterically modulate the function of M_1_R and possibly other G protein-coupled receptors.

## 1. Introduction

Bile acids are amphipathic hydroxylated steroid byproducts of hepatic cholesterol metabolism that act as detergents which facilitate lipid absorption by emulsifying dietary fats. Over the past three decades, the known role of bile acids has expanded beyond lipid absorption to recognize their functions as signaling molecules that interact with both cell membrane (e.g., TGR5) and nuclear (e.g., FXR) receptors [1,2,3]. The present research focus was instigated by our observations that bile acids interact functionally with muscarinic receptors expressed by guinea pig gastric chief cells [4] and human colon cancer cells [5]. Our later finding that bile acids can activate cardiovascular muscarinic receptors [6] was confirmed by other investigators who reported that taurocholate acts as a partial agonist for neonatal rat cardiomyocyte M_2_ muscarinic receptors, thereby reducing levels of intracellular cAMP and slowing the heart rate [7]. Bile acids have also been reported to interact functionally with muscarinic receptors in the lungs [8].

Cholinergic muscarinic receptors in the Class A G protein-coupled receptor family are widely expressed in mammals and play key roles in physiology and disease [9,10]. Of the five muscarinic receptor subtypes, designated M_1_R-M_5_R and encoded by *CHRM1*–*CHRM5*, the M_1_R and M_3_R receptor subtypes are highly expressed by intestinal epithelial cells and modulate absorption, secretion, and other important homeostatic processes. We and others demonstrated that M_3_R/*CHRM3* overexpression by gastrointestinal cancers plays a functional role in tumor progression [11]. In contrast to the actions of M_3_R as a colon cancer promoter, M_1_R appears to function as a tumor suppressor. Strong evidence supports the latter claim—M_1_R deficiency in murine colon cancer models augments colon neoplasia [12], increasing concentrations of M_1_R agonists progressively reduce human colon cancer cell proliferation [13], and, compared to adjacent normal colon, M_1_R/*CHRM1* expression is significantly reduced in human colon cancers [14].

Regarding the clinical implications of putative functional interactions of bile acids with cholinergic muscarinic receptors, there has long been an interest in deciphering the role of diet and gut constituents, particularly bile acids [15], as risk factors for the development and progression of gastrointestinal cancer. In this regard, the observation that feeding selected bile acids to mice can induce colon neoplasia is noteworthy [16,17]. Nonetheless, despite these interesting findings, a precise molecular basis underlying the interaction of bile acids with muscarinic receptors has been elusive. Our research has focused on filling this key knowledge gap.

Our discovery more than 27 years ago that bile acids interact functionally with muscarinic receptors [4] provided the basis for a potentially informative line of investigation. Although preliminary insights using receptor modeling to understand the structural basis for the interaction between bile acids and muscarinic receptors were instructive [18], we were puzzled by disparate results in different biological systems. Whereas atropine blocked the interaction of lithocholytaurine with guinea pig gastric chief cells [4], in other cell types, non-muscarinic receptor subtype-selective antagonists inconsistently attenuated bile acid effects. This suggested several possibilities; bile acids might not be competing with muscarinic receptor antagonists at orthotopic binding sites and/or bile acids could be interacting differently with different muscarinic receptor subtypes. Thus, to explain these discordant findings, we hypothesized that bile acids might alter muscarinic receptor activity by interacting with allosteric binding sites on specific muscarinic receptor subtypes. Whereas the potential for allosteric modulation of muscarinic receptor activity is not a novel concept [19], the recent finding of allosteric binding of cholesterol to M_1_R [20] provided conceptual support for our hypothesis. As derivatives of cholesterol metabolism in the liver, bile acids share many structural features with the parent molecule [21]. Hence, it was plausible that some, if not most, functional interactions between bile acids and muscarinic receptors could be explained by their allosteric binding to M_1_R.

In the present work, we used molecular modeling [22,23] to explore binding of bile acids and their glycine and taurine conjugates to M_1_R. We provide in silico evidence that both primary and secondary bile acids can bind to the same allosteric site between TM5 and TM6 on M_1_R as suggested for cholesterol in a previous work as well as to another allosteric site identified between TM3 and TM4. Moreover, we predict that bile acid conjugation with glycine or taurine augments allosteric binding interactions. We also analyzed the effects of bile acid binding to these predicted allosteric binding sites on M_1_R activation.

## 2. Materials and Methods

The full computational pipeline that was conducted in the current study is illustrated in the Supporting Information (SI) Scheme S1. The workflow includes site identification by ligand competitive saturation (SILCS) simulation [24] to map fragment binding patterns on M_1_R; SILCS-Hotspots analysis [25] to predict allosteric binding sites of bile acids; SILCS-Monte Carlo (SILCS-MC) docking [24] to dock acetylcholine (ACh) to the active site or bile acids to the identified allosteric binding sites; molecular dynamics (MD) simulations on the binary system of M_1_R-ACh and allosterically modulated complex of M_1_R-ACh-bile acids to model their dynamics; and final analyses on the MD trajectories to explore the allosteric modulations of bile acids on M_1_R. Computational details are described below for each component. Chemical structures of studied molecules can be found in Supporting Information Diagram S1.

### 2.1. SILCS Simulations

SILCS simulations were conducted using the previously described protocol [24] to map fragment binding affinity patterns on the M_1_R protein. The crystal structure of the inactive form of the M_1_R protein was obtained from the protein data bank (PDB) [26] under entry 5cxv [27] with the antagonist molecule, cholesterols, lipids, and water removed, and then processed using the CHARMM-GUI server [28,29] to set up the simulation system. The protein was placed in a 1-palmitoyl-2-oleoyl-sn-glycero-3-phosphocholine (POPC) lipid bilayer to represent the membrane environment. Then 10 individual simulation systems were generated and simulated independently with each system including, in addition to the protein and bilayer, explicit water and eight randomly positioned types of solutes including benzene, propane, methanol, formamide, dimethyl ether, imidazole, methylammonium, and acetate at approximately 0.25 M each. The SILCS simulations were conducted in a set of steps composed of energy minimization, equilibration, and production runs using iterative MD/grand canonical Monte Carlo (GCMC) [30] cycles to propagate the trajectories, resulting in 20 million steps of GCMC and 100 ns MD production time per simulation, yielding a cumulative total of 200 million steps of GCMC and 1000 ns MD production time over all 10 systems. Detailed system setup and simulation methods were described in our previous work [24]. The GCMC simulations were run using the SILCS software suite, version 2023 (SilcsBio LLC, Baltimore, MD, USA), and MD simulations were performed using GROMACS (Version 4.6) [31,32], with the protein, solutes, and water being described using the CHARMM36m protein force field [33], the CHARMM General force field (CGenFF) [34], and the TIP3P water model modified for the CHARMM force field [35], respectively.

After SILCS simulations, fragment occupancy maps (FragMaps) were generated by binning selected solute or water atoms into voxels on a 1 Å spaced grid spanning the simulation system and were combined to obtain both specific and generic FragMap types as previously described [24]. The FragMaps were then normalized according to fragment bulk values under the absence of protein and were Boltzmann transformed to free energies for each functional group type to yield grid free energy (GFE) FragMaps. Four generic FragMaps were generated, including hydrophobic (APOLAR), heterocycle carbon (GEHC), hydrogen-bond donor (HBDON), and hydrogen-bond acceptor (HBACC) maps, as described previously [24]. In addition, four specific FragMaps were generated, including positive MAMN (methylammonium nitrogen) maps, negative ACEC (acetate carboxyl carbon) maps, alcohol MEOO (methanol hydroxyl oxygen) maps, and formamide carbon (FORC) maps. These FragMaps represent functional group affinity pattern-defining interactions with the protein and were used to guide the identification of allosteric binding sites and docking of bile acids to the M_1_R protein. An exclusion map was also produced which describes the solute and water-forbidden regions during the SILCS simulation, thereby accounting for protein flexibility, and was used as an alternative to the protein solvent-accessible surface in the docking calculations.

### 2.2. SILCS-MC Docking

SILCS-MC docking was conducted to predict binding modes of ACh or bile acids to the M_1_R protein. Targeting the SILCS FragMaps and the exclusion map, SILCS-MC adopts the Monte Carlo sampling method [36] to sample binding orientations and conformations of a ligand. Energy used in the Metropolis criteria relies on atomic GFE scores of selected atoms in each ligand based on a rule file that links CGenFF atom types to SILCS FragMap types, with the atomic GFE scores summed into a ligand GFE (LGFE) that is the predicted binding strength of a ligand [24]. The LGFE score along with the CGenFF intramolecular energy are combined and used as the basis of the Metropolis criteria. Non-hydrogen atoms overlapping the exclusion map are assigned a GFE value of 1000 kcal/mol that is included in the LGFE score. For the current work, SILCS-MC was conducted under an exhaustive mode where ligand conformations were sampled in the FragMaps and exclusion map by allowing the MC translational, rotational, and dihedral variations of up to 1 Å, 180 degrees, and 180 degrees at each MC step. After 10,000 steps of MC conducted at 300 K, a second round of local MC was then performed for 10,000 steps with translational, rotational, and dihedral variations of up to 0.2 Å, 9 degrees, and 60 degrees at the same temperature to further converge the sampling results. Simulated annealing (SA) [37] was then performed for 40,000 steps with the temperature gradually cooled down from 300 to 0 K with translational, rotational, and dihedral variations of up to 0.2 Å, 9 degrees, and 9 degrees to converge the sampling to a local free energy minimum. Each cycle of two MC runs and one SA run is performed up to 1000 times, with the energy difference among the top three binding poses checked every 100th cycle. If the difference is less than 0.03 kcal/mol, then the sampling is treated as being converged. The lowest energy conformation based on the LGFE is selected to represent the binding mode prediction.

### 2.3. SILCS-Hotspots Analyses

SILCS-Hotspots [25] calculations were conducted using SILCS FragMaps and SILCS-MC docking to globally explore potential binding sites of bile acids on the M_1_R protein surface. The 3D space encompassing the full protein was divided into multiple cubic blocks, and exhaustive SILCS-MC docking was conducted for all the bile acids in each subspace with docking poses and LGFE values collected for the following clustering step. Docking poses were first clustered with respect to each bile acid to identify local binding sites. This was followed by a second round of clustering over all the bile acids to identify common binding sites. Clustering involved identifying cluster centers defined as the centroid of a ligand binding pose with the most neighbors. Following the second rounds of clustering over all the bile acids, the average LGFE score was calculated over all the bile acids in each cluster. This score was used to rank the clusters, and the highly ranked hotspot clusters were utilized to identify putative bile acid binding sites and were validated through conventional MD simulations as described in the following.

### 2.4. MD Simulations of ACh Bound Complexes with and Without Bile Acids

MD simulations were initiated from the crystal structure of the inactive M_1_R protein (PDB ID: 5cxv) [27]. Each system included the agonist ACh at the orthosteric site obtained from SILCS-MC docking along with the best docking poses of each bile acid for the considered allosteric binding sites. The system design was selected to investigate the impact of bound bile acids on the conversion from the inactive to the active conformation. The complex structure was processed through the CHARMM-GUI server [28,29] with the membrane environment modeled using a POPC lipid bilayer, following which water and Na^+^ and Cl^−^ ions were added, with ions at a concentration of 0.15 M. Each system was then subjected to energy minimizations followed by six rounds of MD equilibration according to the CHARMM-GUI equilibration protocol for membrane systems [38]. It should be noted that, similar to previous modeling works on muscarinic receptor proteins [39,40], we used a simplified lipid bilayer setup based on a single lipid type, POPC. Physiological membrane compositions are much more complex and joint effects from bile acid, cholesterol, and lipids could be further explored using various physiological membrane setups. Following the MD equilibration rounds, production runs were conducted in the NPT ensemble without any restraints for 1.5 μs with a 2 fs time step with periodic boundary conditions at a system temperature and pressure of 303 K and 1 atm, respectively. This temperature and pressure were controlled by the Nosé–Hoover thermostat [41,42] and Parrinello–Rahman barostat [43]. The non-bonded interactions were computed with a cutoff of 12 Å using the Verlet cutoff scheme [44]. A switching function [45] was applied for Lennard-Jones (L-J) interactions from 10 to 12 Å along with the long-range isotropic dispersion correction [46] to the energy and pressure for L-J interactions beyond 12 Å. The long-range electrostatic contribution is handled by the particle mesh Ewald (PME) method [47] with a real space cutoff of 12 Å. The same set of force fields as described above for SILCS simulation was adopted and GROMACS was used to conduct all MD simulations.

### 2.5. Analyses of MD Trajectories

Production run MD trajectories were analyzed using various GROMACS utilities [31,32] to extract different dynamical and structural information. Visualization of MD trajectories was performed through VMD software (Version 2.0.0) [48]. Linear mutual information (LMI) [49] analyses were conducted using the correlationplus python package [50], which was also used to make the correlation plots.

## 3. Results

### 3.1. Functional Group Binding Patterns Mapped for M_1_R

SILCS was used to identify potential bile acid binding sites on M_1_R. SILCS is a co-solute GCMC/MD-based methodology [24] that offers a rigorous free energy binding computational assay of utility for various molecular analyses and drug design needs [51]. In the current study, we used SILCS to predict allosteric binding sites as well as binding poses of bile acids for M_1_R.

**Figure 1:**
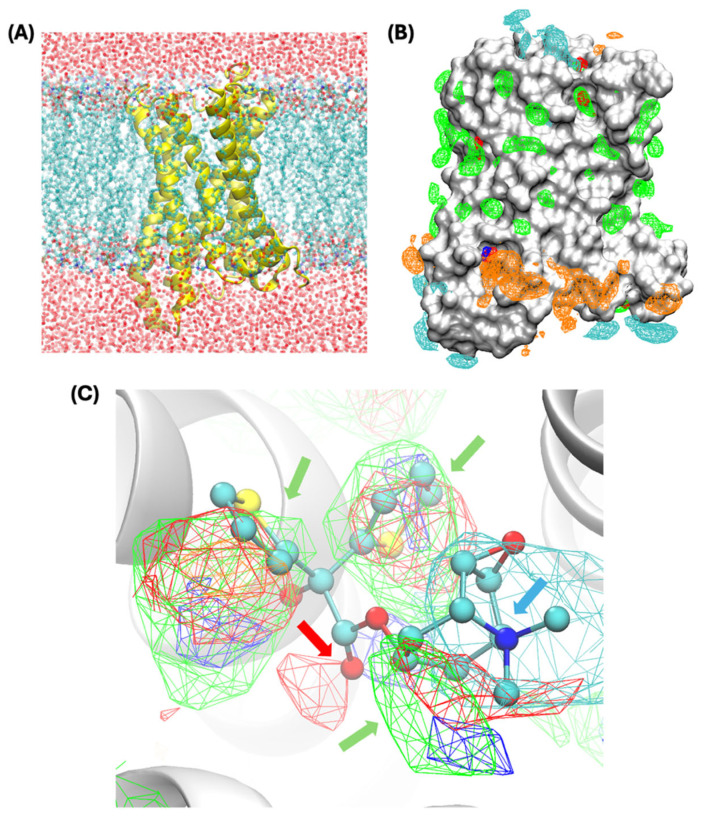
Simulation system and SILCS FragMaps surrounding the full protein and in the active site. (**A**) The main simulation system used for the current study. The M_1_R protein (yellow) is embedded in a lipid bilayer composed of POPC molecules (shown in ball and stick representation with carbons in cyan); intra- and extracellular regions were modeled using water (only oxygen atoms are shown in red) and Na^+^ and Cl^−^ ions (not shown). (**B**) SILCS FragMaps for the M_1_R protein are shown for apolar (green), hydrogen-bond acceptor (red) and donor (blue), and negative (orange) and positive (cyan) functional group classes rendered at GFE levels of −1.0, −1.0, −1.0, −1.2, and −1.2 kcal/mol, respectively. The protein is shown in the surface representation. (**C**) Zoomed-in view of FragMaps at the orthosteric site aligned with the crystal binding orientation of the antagonist Tiotropium. Protein is shown in the cartoon representation. FragMaps are rendered with the same levels and colors as in panel B. Consistency of the FragMap types and the chemical groups in the antagonist are indicated by arrows using colors consistent with the FragMap type color code.

The system configuration is illustrated in Figure 1A, showing the M_1_R protein embedded in a lipid bilayer with aqueous solution occupying the intra- and extracellular regions. Solute molecules are then added in the SILCS simulation to map functional group affinity patterns, termed FragMaps, of the M_1_R protein. The SILCS FragMaps are shown in Figure 1B,C. As shown in Figure 1B for the entire protein, the pattern of the FragMap types corresponds to the lipid bilayer chemical environment in the central region of the protein. As shown in the green-colored meshes, apolar FragMaps are mainly found around the region of the protein interacting with the lipid bilayer while H-bond donor or acceptor or charged FragMaps are found accumulated around the intracellular and extracellular regions corresponding to the aqueous environment. Convergence of the FragMaps was verified based on the overlap of the FragMaps (overlap coefficient >0.7) from SILCS simulation runs 1–5 and 6–10 as previously discussed [24]. As additional validation for the predicted FragMap patterns for M_1_R, the crystal binding mode of the antagonist Tiotropium from the inactive M_1_R structure [27] used for all simulations is aligned with SILCS FragMaps at the orthosteric site (Figure 1C). The binding of the antagonist Tiotropium involves contacts between aromatic thiophene rings as well as the aliphatic nonane ring in the ligand and the hydrophobic characteristics formed by the tyrosine cage residues Y131, Y430, Y453, and Y457 as well as other surrounding aromatic or aliphatic residues such as W182, L208, and A221 [27]. Hydrogen bonding interaction between the central carbonyl group in the ligand and residue N382 and charged interaction between the quaternary ammonium ion in Tiotropium and residue D105 were also well defined. As indicated by the arrows in Figure 1C, the FragMaps at the orthosteric site reproduced important crystal binding modes of the antagonist Tiotropium, with the apolar FragMaps aligning well with the two thiophene rings, as well as the azatricyclo [3.3.1.0^2,4^]nonane ring in the ligand and H-bond acceptor map aligning with the central carbonyl group in the ligand, and the positively charged map aligning with the quaternary ammonium ion. This validates that SILCS FragMaps accurately predicted the requirements of ligand chemical types that can be accommodated within the orthosteric site of M_1_R for the particular antagonist under consideration. In addition, FragMaps also captured binding patterns that are not utilized by the current antagonist, for example, neutral H-bond donor map near the quaternary ammonium moiety which could be occupied by other antagonists, agonists, and bile acids and used to predict their binding poses.

### 3.2. Prediction of Allosteric Binding Sites and Binding Poses of Bile Acids

A previous work [52] studied the allosteric effect of cholesterol binding on the activation of muscarinic receptor M_1_R. The work showed that binding of cholesterol slowed receptor activation. Here, we investigate the possible allosteric effect of the binding of bile acids to M_1_R.

**Figure 2:**
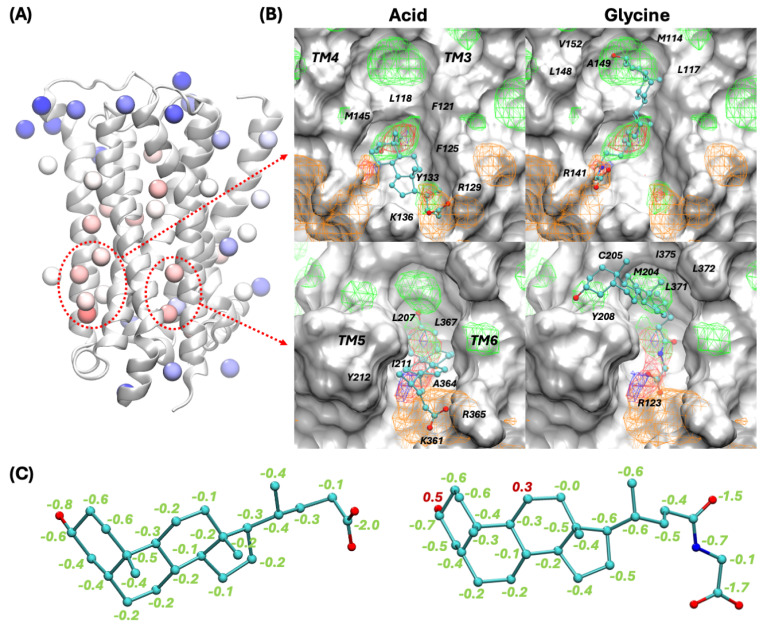
SILCS-Hotspots analysis and predicted docking poses of a representative bile acid and its glycine conjugate at the two top-ranked binding sites. (**A**) SILCS-Hotspots showing the predicted bile acid hotspot sites. Binding cluster centers are shown in spheres and colored in the red–white–blue scale for LGFE scores, with red indicating more favorable LGFE values. The two top-ranked binding sites are indicated by red dashed circles (AS34 on the left and AS56 on the right). (**B**) Docking poses of lithocholic acid and its glycine conjugate at the two predicted binding grooves. The protein surface and SILCS FragMaps at the same rendering level as Figure 1 are shown. Important binding site residues are labeled. (**C**) Atom GFE contributions to the total LGFE score for lithocholic acid and its glycine conjugate at the AS34 binding groove.

The SILCS-Hotspots workflow [25] was employed to predict potential allosteric binding sites of bile acids as well as their corresponding glycine and taurine conjugates (Table 1). Figure 2A shows the SILCS-Hotspots results with identified cluster centers (i.e., hotspots) on the protein structure and colored according to their LGFE scores. As indicated by the dashed circles, the top-ranking hotspots based on average LGFE scores are found in two regions. One is near a groove formed by intracellular halves of helices TM3 and TM4 (defined as AS34) and the second is between helices TM5 and TM6 (named AS56) on the intracellular side, respectively. The AS56 allosteric binding site resembles the previously proposed allosteric binding site for cholesterol [52].

The predicted binding mode of lithocholic acid as well as that of its glycine conjugate for sites AS34 and AS56 are shown in Figure 2B. The binding modes of the other molecules can be found in Figures S1 and S2 in the Supporting Information. Binding of lithocholic acid at the AS34 site is predicted to be stabilized by the steroid skeleton’s contacts with the hydrophobic environment created by residues L118, F121, F125, Y133, and M145, as also indicated by the apolar FragMaps shown in green meshes. Salt bridge interactions between the tail carboxylate group in lithocholic acid and basic residues R129 and K136 are also present, as indicated by the negatively charged FragMaps shown as orange meshes. For site AS56, hydrophobic contacts between the steroid skeleton in bile acids and neighboring residues L207, I211, Y212, A364, and L367, as well as salt bridge interactions between the carboxylate tail and K361 and R365, are seen. Similar binding modes for other bile acids at the two predicted allosteric binding sites are observed, indicating the consistency of bile acid binding to M_1_R. With increasing tail length, the predicted binding poses of glycine and taurine conjugates can go deeper into the lipid bilayer region and have contacts with more hydrophobic residues at the top of the binding groove. For example, lithocholylglycine can have hydrophobic contacts with more residues such as M114, L117, L148, A149, and V152 at the AS34 site, and M204, C205, Y208, L371, L372, and I375 for the AS56 site. And the carboxylate tail can form a salt bridge interaction with alternative basic residues on the intracellular side, such as R141 for the AS34 site and R123 for the AS56 site. Again, similar binding modes for other bile acid conjugates are seen as presented in the Supporting Information.

Table 1 lists the predicted binding affinities in terms of LGFEs for all ligands considering both allosteric sites. Predicted binding affinities are similar across different types of bile acids for the individual sites. Upon conjugation with glycine and taurine, binding is generally enhanced. Comparing the two identified allosteric binding sites, all bile acids as well as their conjugates favor site AS34 over AS56. Figure 2C shows the atomic GFE contributions of each classified atom to the overall predicted binding affinity in terms of LGFE for lithocholic acid. The aliphatic atoms throughout the steroid skeleton each make small favorable contributions consistent with their overlap with the apolar FragMaps, making the largest overall contribution to binding. Favorable contributions are also observed from the polar atoms in the sidechains, with the individual acid groups making substantially favorable contributions. GFE contribution analyses for other molecules can be found in Table S1; similar distributions for the different functional groups are seen. Regarding glycine and taurine bile acid conjugates, the introduction of glycine and taurine groups allows the molecule to achieve an ideal size to occupy other hydrophobic subsites in the binding groove while maintaining other favorable polar interactions and leading to significantly enhanced overall binding affinities. For example, for lithocholylglycine, shown in the right panel in Figure 2C, the glycine portion contributes −4.0 kcal/mol GFE in total vs. −2.0 kcal/mol for the carboxylate group alone without conjugation. To compare our findings to the previous work on cholesterol, we also docked cholesterol to the two binding sites, yielding LGFE values for the AS34 and AS56 sites of −10.68 and −7.10 kcal/mol, respectively, which are comparable to our predicted bile acid binding affinities. Moreover, bile acid glycine or taurine conjugates are predicted to bind more tightly to M_1_R at both allosteric sites than cholesterol. In addition to the docking results, in the future, MD simulations on systems containing both bile acid and cholesterol could be explored to study their competition and their joint effects on modulating M_1_R.

### 3.3. Lithocholic Bile Acids Stay in the Two Predicted Binding Sites Through Microsecond MD on the M1R-ACh-Bile Acid Complexes

MD simulations were undertaken to verify the stability of the lithocholic bile acids in the binding sites identified by SILCS-MC and their potential allosteric impact on receptor activation. The simulations were initiated from the inactive M_1_R crystal structure [27] with the physiological muscarinic receptor agonist ACh docked to the orthosteric binding site using SILCS-MC. The simulation setup was selected based on the assumption that the bile acids will bind to and stabilize the inactive form of M_1_R as they are negative allosteric modulators even in the presence of an agonist. This setup also allows for the impact of the bile acids on conformational transitions between the inactive and active forms to be monitored, as presented in Section 3.4 below. The three lithocholic molecules, including both their bile acid and conjugate forms, were selected based on their highly favorable SILCS-MC LGFE scores (Table 1), with the docked orientations used to initiate the simulations. This includes simulations of the M_1_R-ACh-bile acid allosterically modulated complexes with the bile acids docked in either of the two top-ranked allosteric binding sites, AS34 and AS56.

**Figure 3:**
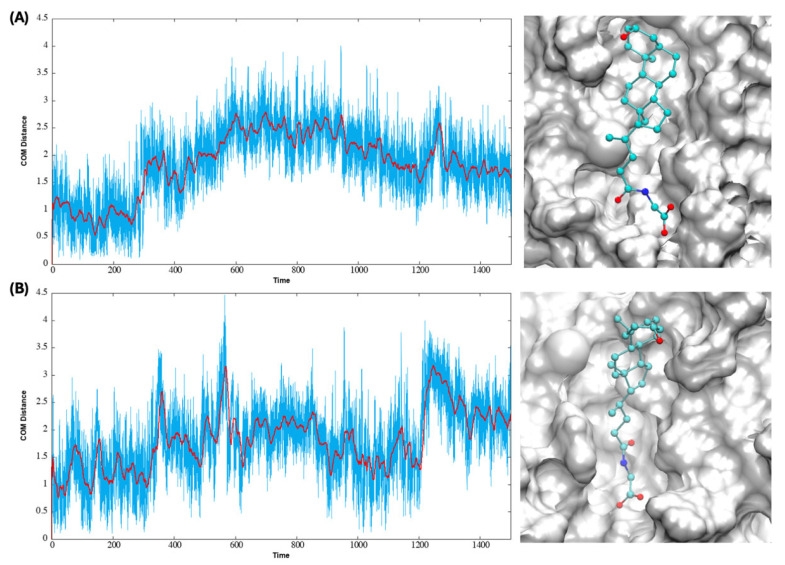
Center-of-mass (COM) distance (in Å) between lithocholylglycine during the MD simulations (time in ns) and its initial docking orientation and the binding poses from the last frame of the MD simulations for sites AS34 (**A**) and AS56 (**B**). Moving average values of distances calculated every 15 ns are also shown as red lines.

Figure 3 plots the distance between the COM of lithocholylglycine during the MD simulation and that of the initial docking pose for both allosteric sites. The relatively stable COM distance values across the simulation, with fluctuations for both sites, indicate the ligand is accommodated well in the binding sites, though with frequent reorientations. The same plots for lithocholic acid and its taurine conjugate can be found in Figure S3. Figure 3 also shows the binding poses of lithocholylglycine from the final frame of the MD simulation for the two sites, and the binding poses are comparable to the docking poses shown in Figure 2B, even though reorientation of the steroid skeleton and carboxylate tail is seen.

**Figure 4:**
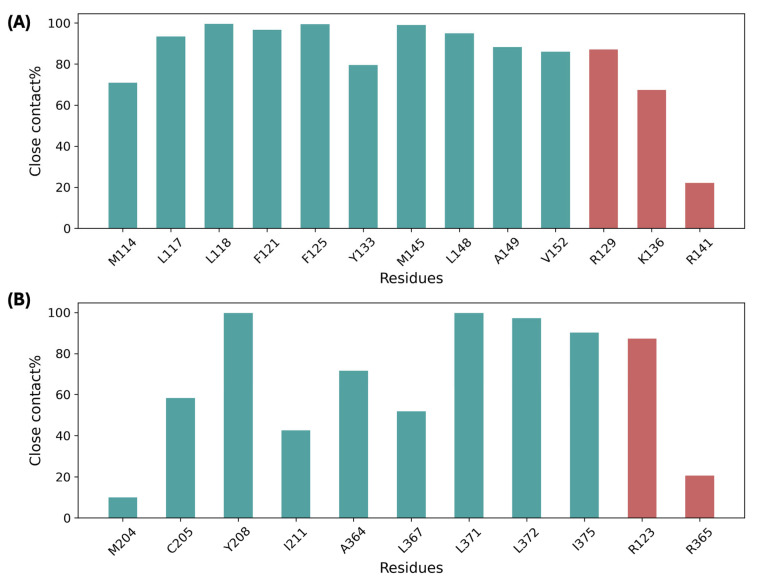
Percentages of close contact formed by lithocholylglycine with binding site residues along the whole MD simulation for sites AS34 (**A**) and AS56 (**B**). Close contact is defined as any non-hydrogen atom in the ligand being within 3 Å of any non-hydrogen atom in the protein residue. Hydrophobic residues are colored in green and basic residues in red.

Figure 4 shows the interaction analysis of lithocholylglycine with binding site residues from the MD simulation. At the AS34 site, lithocholylglycine is in close contact with the selected hydrophobic residues on TM3 and TM4 shown in Figure 2B for most of the simulation time, with close contacts with residues L118, F125, and M145 for almost 100% of the simulation time. Close contacts of lithocholylglycine with basic residues are also seen associated with the carboxylate tail, potentially forming a salt bridge interaction with those residues, especially R129. Similarly, for the AS56 site, the ligand stays close to most of the labeled hydrophobic residues on TM5 and TM6, with close contacts formed with residues Y208 and L371 for almost all the simulation time. Again, close contacts between the ligand with basic residues in the binding site are seen, with R123 being the most frequent residue in contact. The results are similar for lithocholic acid and taurine conjugate and can be found in Figure S4. The relatively stable binding of bile acids observed through the microsecond MD simulations further validates the two identified allosteric binding sites for bile acids. The predicted interacting residues with bile acids at the allosteric sites could help to design mutagenesis studies to experimentally validate such bile acid binding predictions. For example, residues F125 and R129 were predicted to serve as key nonpolar and polar interacting residues, respectively, with lithocholylglycine at the AS34 site. Accordingly, site-directed mutations such as F125A and R129Q could be employed to experimentally validate bile acid binding and the associated allosteric effects predicted for this site.

### 3.4. Transition Toward the Active State upon Agonist Binding with and Without the Presence of Bile Acids

Given the experimental evidence that bile acids and cholesterol act as negative allosteric modulators of M_1_R, the allosterically modulated complex MD simulations were analyzed to observe the impact of the presence of selected bile acids on the transition from the inactive to the active conformation. As a control, a simulation of the M_1_R-ACh binary complex was performed to determine the extent of conformational change from the inactive form toward the active form in the absence of the selected bile acids.

Previous structural studies on activation of muscarinic receptors show multiple important conformational changes between inactive and active states. These include the global conformational changes involving the outward movement of TM6 and inward movement of TM5 on the cytoplasmic end for the active state and the residue interaction network on the cytoplasmic end undergoing large changes upon activation [39,40,53,54]. The ion lock defined as the salt bridge between two charged residues on TM3 and TM6 (TM3:R123-TM6:E360 for M_1_R), which is present in the inactive state, is broken in the active state. Formation of a hydrogen bond between two tyrosine residues on TM5 and TM7 (TM5:Y208-TM7:Y418 for M_1_R) involving the NPxxY motif is also observed for the active state. Another conformational indicator for the active state is the DRY motif in TM3, as previously reported [39,40]. The DRY motif includes R123, which is involved in the ionic lock mentioned above.

**Figure 5:**
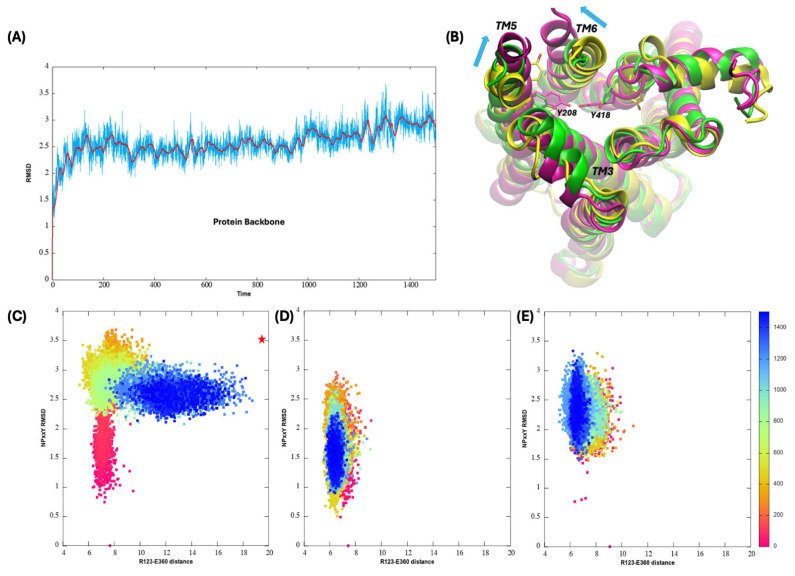
Analyses of the MD trajectories for the M_1_R-ACh binary complex and the allosterically modulated complex M_1_R-ACh-lithocholylglycine. (**A**) RMSD in Å of protein backbone atoms during the M_1_R-ACh binary complex production MD simulation, with time shown in ns. Moving average values of RMSD calculated every 15 ns are also shown as red lines. (**B**) Comparison of active (PDB: 6oij in purple) and inactive (PDB:5cxv in yellow) crystal structures as well as the last frame protein structure (green) from MD on the binary complex. Residues Y208 and Y418 are shown. (**C**) Two-dimensional plot of COM distance between residues R123 and E360 versus the RMSD of the NPxxY motif with reference to the inactive conformation along the MD simulation on the binary complex. The red star symbol indicates the values from the crystal structure of the active M_1_R. The same two-dimensional plots from MD simulations on the allosterically modulated complex M_1_R-ACh-lithocholylglycine are also shown for both the AS34 site (**D**) and the AS56 site (**E**) binding of the bile acid glycine conjugate.

Analysis of the MD simulations focused on the conformational changes occurring during the MD simulations as well as on the binding orientation of ACh, which was shown to remain in the orthosteric site (Figure S5). Figure 5A shows the root-mean-square deviation (RMSD) of the protein backbone atoms during the M_1_R-ACh binary complex MD simulation, and a climbing trend is observed which indicates conformational changes are happening associated with the activation process. In Figure 5B, the final structure from the 1.5 µs MD simulation is aligned with both the inactive (PDB entry:5cxv) [27] and the active (PDB entry:6oij) [55] crystal structures of M_1_R. The outward tilting of TM6 and inward tilting of TM5 on the cytoplasmic end compared to the inactive structure are evident, though the changes have not reached the full active conformation. The two tyrosine residues in the final frame of the MD simulation are much closer to each other when compared to the original inactive structure but are still blocked by L367 and have not yet formed a hydrogen bond.

To quantify the inactive to active conformational change during the MD simulation, we tracked the RMSD of the NPxxY motif and the distance between residues R123 and E360 involved in the ionic lock. A two-dimensional analysis of the RMSD of the NPxxY motif versus the COM distance between the two ion lock residues R123 and E360 as a function of simulation time is shown in Figure 5C. The figure includes the values observed in the active and inactive crystal structures. As can be seen, in the M_1_R-ACh binary complex simulation, the two descriptors shift from their positions in the inactive state (starting structure as indicated by the red point on the X-axis) toward those of the active state (indicated by the red star symbol) by the end of the simulation. This includes the distortion of the NPxxY motif as well as the separation of the ion lock residues, indicating the binary complex MD simulation captures, to a large extent, conformational changes associated with activation of M_1_R upon the binding of agonist ACh. The distortion of the NPxxY motif happens very quickly and it reached a stable conformation around 0.8 µs, following which the separation of the two ionic lock residues began to occur. To fully observe the transition to the active conformation, a longer MD simulation or MD simulations with enhanced sampling—for example, the accelerated MD method as used previously [39,40]—would be required, but since our focus was to study the potential allosteric effects of bile acids, the extent of the conformational change that occurred during the 1.5 µs conventional MD simulation was deemed appropriate.

Figure 5D,E show the two geometric metrics, the COM distance between R123 and E360 versus the RMSD of the NPxxY motif as a function of the simulation time for the allosterically modulated complex M_1_R AS34- and AS56-bound lithocholylglycine systems. As compared to the bile acid-absent scenario shown in Figure 5C, despite distortions of the NPxxY motif, short distances between the two ionic residues were maintained for both the AS34- and AS56-bound lithocholylglycine systems in the µs-long MD simulations. This indicates that the conformational transition from inactive to active state caused by binding of ACh to the orthosteric site was hindered upon binding of lithocholylglycine to both suggested allosteric binding sites on M_1_R. As shown in Figure S6, similar trends were observed for lithocholic acid and lithocholyltaurine.

### 3.5. Activation Mechanism of M_1_R upon ACh Binding

Previous studies of other muscarinic receptor proteins proposed multiple activation mechanisms with different levels of atomic detail [39,54]. Miao et al. applied the dual-boost accelerated MD method to study M_2_R activation [39]. Starting from an inactive conformation in the absence of the antagonist, they sampled broad collections of conformations of the apo protein and suggested an allosteric activation pathway that contains two typical intermediate states. During the transition from the inactive state to intermediate state 1, active site residues W400 on TM6 and Y430 on TM7, as well as residue Y440 on the TM7 intracellular side, underwent conformational changes. On the path from the intermediate state 1 to 2, Y206 on the TM5 intracellular side reorients its sidechain from the initial position between TM3 and TM6 to the lipid-exposed side of TM6. During the final transition to the active state, Y206 and Y440 relocate their sidechains toward each other to form a hydrogen bond. The study found that W400 is a key residue acting as a conformational switch, consistent with the transmission switch role of this conserved tryptophan residue found for adenosine receptors [39]. The study also emphasized the role of active site residue Y430, which is involved in a key conformational change involving its relocation from the active site.

In another study on M_2_R, Gong et al. used MD simulations to study the activation process due to the agonists ACh and Iperoxo [54]. In that work, they focused on the conformational properties of residues on TM5 and TM6 and suggested a detailed conformational coupling pathway in transducing signals across the membrane from the active site to the intracellular side. Again, the transmission switch role of W400 was confirmed and its coupling with F396 and following sequential conformational dynamics through residues L393 and I389 along TM6 were suggested.

The trajectory from the present MD simulation on the binary M_1_R-ACh complex was examined frame-by-frame, and key conformational changes related to the activation were identified. The orthosteric site is composed of the so-called tyrosine cage formed by Y106 on TM3, Y381 on TM6, and Y404 and Y408 on TM7, along with W378 on TM6 and D105 on TM3. Upon ACh binding, the interaction network will affect the orthosteric site residue dynamics, leading to conformational changes down the TM helices. Three important sequential conformational changes that can be linked to activation were observed in the MD trajectory. First, there was distortion of the NPxxY motif, especially conformational changes in Y418 and its hydrogen bonding network. Second, disruption of the ionic lock caused by the structural dynamics of W378 as well as Y418 involved hydrogen bonding interactions with R123. Third, the structural dynamics of residue Y208 changed. These observations for M_1_R are consistent with previous findings for M_2_R but differ in atomic details. Detailed analysis can be found in the SI, and it should be noted that this observation is based on a single trajectory conducted in our study as a control for other allosterically modulated complex MD simulations; multiple MD simulations could be conducted on the binary system to further validate such observations.

### 3.6. Bile Acid Binding Stabilized the Allosteric Sites and Other TM Regions

**Figure 6:**
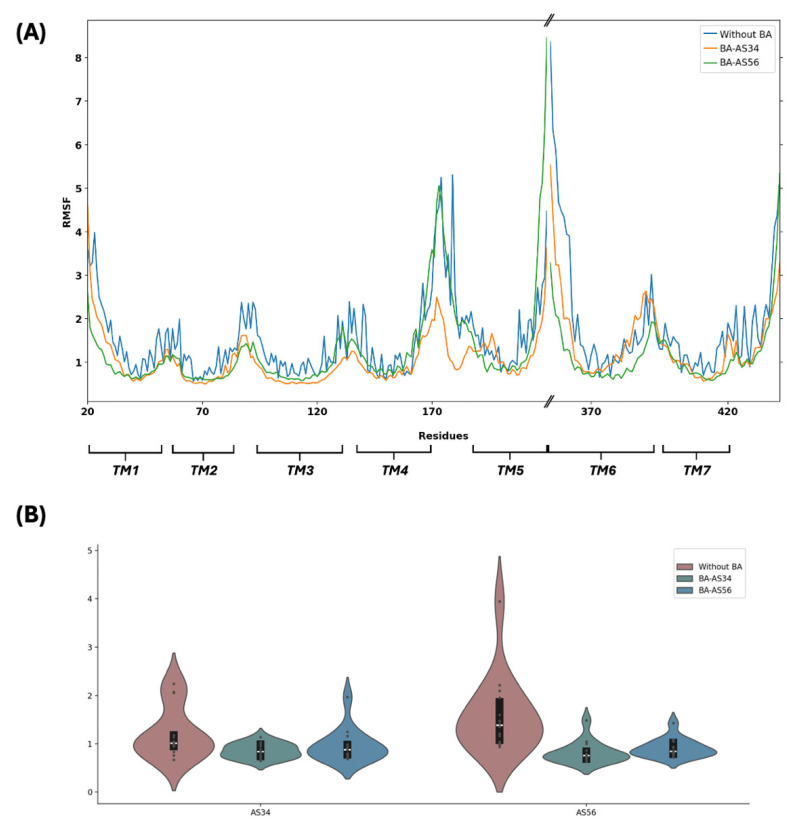
Root-mean-square fluctuation analyses of MD simulations with and without bile acid. (**A**) RMSFs of all residues in M_1_R through MD simulations of M_1_R-ACh and M_1_R-ACh-lithocholylglycine considering both the AS34 and AS56 sites. (**B**) Distribution of RMSF values of key residues at sites AS34 and AS56 as defined in Figure 2B. The mean value is indicated as the white horizontal line and the black bar defines the third and first quartiles.

To further explore how bile acid allosteric binding dampens the activation of M_1_R, we analyzed residue flexibility in terms of root-mean-square fluctuation (RMSF). Figure 6A shows the RMSF values of all residues from MD simulations on M_1_R-ACh, compared with simulations performed on M_1_R-ACh-lithocholylglycine complexes considering both the AS34 and AS56 sites. In general, when compared to simulation without the bile acid, less residue flexibility throughout the full protein is observed for both AS34- and AS56-bound lithocholylglycine. As expected, the AS34-bound system shows the lowest RMSF values for the TM3 and TM4 regions among the three simulations while the AS56-bound system shows smaller RMSF values for most parts of TM5 and TM6 than the other two simulations. Similar trends are observed for lithocholic acid and its taurine conjugate systems, as shown in Figure S7. Figure 6B shows the RMSF distributions focusing on allosteric site residues for both the AS34 and AS56 sites. Compared to the bile acid-absent case, the more concentrated distributions toward smaller RMSF values for both AS34- and AS56-bound systems at both sites AS34 and AS56 indicate that bile acid binding stabilized the allosteric sites.

### 3.7. Allosteric Site Stabilization Weakened Cooperativity Between TM Domains

**Figure 7:**
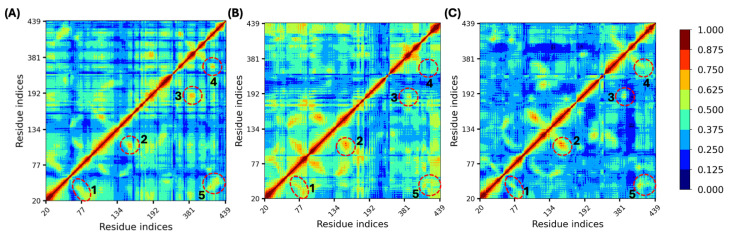
Linear mutual information residue correlation plots calculated from MD simulations. (**A**) LMI for the M_1_R-ACh complex and the M_1_R-ACh-lithocholylglycine complex targeting both the (**B**) AS34 and (**C**) AS56 allosteric sites. Five off-diagonal regions with high LMI values identified for M_1_R-ACh complex simulation are indicated by red dashed circles. Detailed interaction information is tabulated in Table 2.

We also analyzed the dynamic couplings between residues in M_1_R in the MD simulation by calculating a linear correlation metric in terms of LMI of protein structure fluctuations [50]. LMI was suggested to be a better metric than other linear correlation metrics such as dynamic cross-correlation, which was adopted in a previous work on muscarinic receptors [39], since it avoids the angular dependency issue [50]. Figure 7A shows the dynamic map of residue LMI correlations, and a medium level of correlations is observed in many places on the off-diagonal regions which indicates strong dynamic couplings between residues from different TM domains across the entire protein during the transition process. Focusing on regions with LMI values larger than 0.625, five off-diagonal regions as indicated by red dashed circles, designated 1–5, are observed and listed in Table 2. These noted regions are related to the correlated motions between residues on TM1 and TM2, TM3 and TM4, TM5 and TM6, TM6 and TM7, and TM1 and TM7, respectively. Such highly correlated motions between TM5 and TM6 and TM6 and TM7 were also observed previously in Miao et al.’s study on the M_2_R activation process [39]. This indicates that the binding of ACh triggered high correlations of motions of different TM domains, and such a strong correlation network facilitated the conformational transition from the inactive to active state.

Figure 7B,C present the LMI correlation plots for the AS34- and AS56-bound lithocholylglycine complexes. For AS34 site-bound simulation, different correlation patterns are seen when compared to the bile acid absence case. For the AS56 site-bound scenario, fewer correlations are observed compared to Figure 7A, suggesting a disruption of the network between TM domains, which may be critical for activation. Focusing on the five highly correlated regions identified in the bile acid-absent system, for both the AS34 and AS56 cases, weakened correlations are observed for off-diagonal areas 3 and 4 that describe correlated motions for TM5-TM6 and TM6-TM7. This indicates that for both the AS34 and AS56 allosteric site-bound lithocholylglycine systems, the dynamic cooperativity between TM5 and TM6 and between TM6 and TM7 observed in the bile acid-absent system is diminished. Similar trends are observed for lithocholic acid and its taurine conjugates, as shown in Figure S8. As discussed above, the proposed activation mechanism suggests that the interaction network between residues on TM5, TM6, and TM7 is essential for facilitating collective conformational changes that lead to activation. Thus, the enhanced allosteric site stability upon bile acid binding reduces the flexibility of various TM domain residues and weakens cooperativity between TM domains, particularly between the TM5–TM6 and TM6–TM7 pairs, ultimately slowing activation.

### 3.8. Allosteric Bile Acid Binding Distorts Agonist ACh Binding

**Figure 8:**
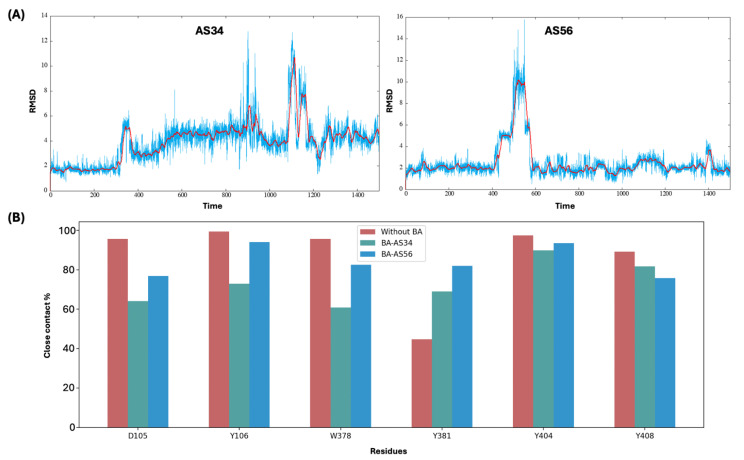
Analyses of ACh binding at the active site. (**A**) RMSD (in Å) of ACh heavy atoms along the MD simulation (in ns) for AS34- and AS56-bound lithocholylglycine systems. Moving average values of distances calculated every 15 ns are also shown as red lines. (**B**) Percentage of close contact formed by ACh with active site residues along the whole MD simulation for M_1_R-ACh and M_1_R-ACh-lithocholylglycine systems for both the AS34 and AS56 sites.

Binding of bile acid at allosteric sites not only degraded cooperativity of TM domains but also distorted binding of agonist ACh at the active site. Figure 8A shows the RMSD plots for non-hydrogen atoms of ACh along the MD simulation time for the AS34 and AS56 allosteric site-bound lithocholylglycine systems. When compared with the RMSD plot for the bile acid absence case as shown in Figure S5, much larger fluctuations are seen for ACh when bile acids are present considering both allosteric sites. Figure 8B shows the close contact percentages of ACh with each key residue that defines the active site through the simulation. When bile acid is absent, ACh has close contacts with multiple residues at the active site for almost the full MD simulation time, especially with D105, Y106, W378, Y404, and Y408. ACh also forms a charged interaction between the quaternary basic nitrogen in ACh and D105 that is crucial for its binding. However, a much-weakened charged interaction between ACh and D105 is observed for both AS34- and AS56-bound lithocholylglycine systems, as indicated by the smaller contact percentage values. Less stable contacts of ACh with most tyrosine residues as well as W378 are also observed for both AS34- and AS56-bound lithocholylglycine cases, though to different extents. Interestingly, enhanced contacts of ACh with Y381 are observed when lithocholylglycine is present. Results for lithocholic acid and its taurine conjugate can be found in Figure S9, and the overall trend of weakened contacts of ACh with active site residues, especially with D105 and W378, is confirmed upon the allosteric binding of various bile acid forms. As discussed above, W378 is a key transmission switch responsible for conformational dynamics on TM6 needed for transition to active conformation; loose contact of ACh with W378 is expected to disrupt the activation process.

## 4. Discussion

In the current work, we employed SILCS and MD simulations to explore bile acid binding to M_1_R. We used SILCS functional group binding patterns on M_1_R to predict allosteric binding sites of bile acids as well as their binding poses. As reported previously for cholesterol, bile acids were predicted to bind to the same allosteric site on M_1_R between TM5 and TM6. We also identified another potential allosteric site, AS34 between TM3 and TM4, which can accommodate the bile acid better than the AS56 site. Showing that lithocholic acid and its glycine and taurine conjugates can stay in the two suggested allosteric sites during the full microsecond MD simulations provides additional proof of concept.

Using SILCS-Monte Carlo simulations, the endogenous agonist ACh was docked to the active site of a M_1_R inactive conformation, and the M_1_R activation process was captured using a 1.5 μs MD simulation. The activation mechanism of M_1_R upon agonist binding determined using the MD trajectory is consistent with the proposed mechanisms for other muscarinic receptors. In parallel, MD simulations on bile acid-bound M_1_R-ACh complexes were conducted with the extracted dynamic conformational information compared to the bile acid-absent scenario. It was observed that bile acid binding slows the activation process of M_1_R and cooperativity between TM domains; binding between TM5 and TM6 and between TM6 and TM7 was especially weakened. These findings suggest that bile acids can allosterically modulate M_1_R activation by enhancing the structural stability of TM3/TM4 or TM5/TM6, leading to a loss of the cooperativity between TM5, TM6, and TM7 needed for activation. Moreover, distortions of ACh binding in the active site observed in MD trajectories when bile acid is present further support the allosteric modulation of M_1_R activity by bile acids.

It should be emphasized that the current work is purely based on computational modeling predictions and needs to be thoroughly validated using wet lab methods, for example, mutagenesis, to validate the allosteric binding sites we propose. It has not escaped our attention that given the broad distribution of muscarinic receptors, including all the segments of the gastrointestinal tract, and that bile acids circulate in the bloodstream, the potential role for bile acids regulating cell function via allosteric effects on M_1_R and other muscarinic receptor subtype signaling in health and disease is exhaustive. Previous studies revealed that bile acid concentrations in the proximal colon are in the high micromolar range, levels that could result in negative allosteric modulation of M_1_R activity and, thereby, alter the function of both normal and neoplastic colonocytes via this mechanism [56].

Considering the structural similarities among muscarinic receptors, it is anticipated that cholesterol and bile acid could share similar allosteric modulating mechanisms with muscarinic receptor subtypes beyond M_1_R. Moreover, after experimental validation, the current in silico findings suggest the possibility that bile acids interact functionally with other G protein-coupled receptors, acting as allosteric modulators. In this context, others highlighted the therapeutic potential of leveraging allosteric interactions to modulate muscarinic receptor activity and function [57]. We plan to explore these prospects in future work. The potential impact of our findings is augmented by the current focus on developing new drugs that selectively stimulate or block M_1_R activation [58] and advances in understanding the role that gut bacteria play in modulating the diversity of human bile acids [59].

## 5. Conclusions

Using the novel SILCS method as well as conventional MD simulations, conformational dynamics of agonist bound M_1_R with and without the presence of bile acids or their glycine and taurine conjugates were fully modeled and analyzed. The simulation observations indicate the potential for unconjugated and conjugated bile acids to serve as negative allosteric modulators of M_1_ muscarinic receptors. In the context of colon cancer, where M_1_R activation is reported to suppress neoplastic cell proliferation, this suggests a novel mechanism whereby bile acids can promote colon neoplasia. More broadly, our finding that allosteric binding of bile acids to G protein-coupled receptors may modulate receptor function is worthy of further exploration.

## Figures and Tables

**Table 1:** LGFE binding affinities (kcal/mol) for all bile acids and their glycine and taurine conjugates at the two predicted allosteric binding sites on M_1_R.

	Acid		Glycine		Taurine	
Name	AS34	AS56	AS34	AS56	AS34	AS56
Cholic	−10.30	−7.41	−11.25	−8.61	−11.33	−9.75
Chenodeoxycholic	−10.64	−7.70	−11.94	−9.94	−11.53	−10.30
Deoxycholic	−9.40	−8.19	−10.99	−9.56	−11.29	−9.95
Lithocholic	−9.91	−7.48	−12.30	−10.29	−12.12	−10.72

**Table 2:** Residue pairs that have high LMI values identified in the five off-diagonal regions as shown in Figure 7.

Off-Diagonal	Domain 1		Domain 2	
Region	TM	Residues	TM	Residues
1	TM1	27/29/31	TM2	83
		37/40		75/76
		39/40/41/43		71/72
2	TM3	105–114	TM4	151–157
		99		161
3	TM5	183–191	TM6	383–390
4	TM6	364–370	TM7	416–421
5	TM1	39	TM7	412
		41–43		414–416
		48/49		425/427/428
		55/56		424/425

## Data Availability

The original contributions presented in this study are included in the article/supplementary material. Further inquiries can be directed to the corresponding author(s).

## Supplementary Materials

The following supporting information can be downloaded at https://www.mdpi.com/article/10.3390/biom15091326/s1: Scheme S1: Flowchart illustrating the computational pipeline conducted in the current work; Diagram S1: Two-dimensional chemical structures of all small molecules studied in the current work; Figure S1: Predicted binding poses for all considered bile acid and glycine and taurine conjugates at the AS34 site using SILCS-MC; Figure S2: Predicted binding poses for all considered bile acid and glycine and taurine conjugates at the AS56 site using SILCS-MC; Table S1: Functional group GFE contributions to LGFE (kcal/mol) for all bile acids and their glycine and taurine conjugates at the two predicted allosteric binding sites on M_1_R; Figure S3: COM distance between the COM of lithocholic acid or lithocholyltaurine during the MD simulations and that of the initial docking orientation and the binding pose from the last frame of the MD simulations for sites AS34 and AS56; Figure S4: Percentage of close contact formed by lithocholic acid or lithocholyltaurine with binding site residues along the whole MD simulation for sites AS34 and AS56; Figure S5: RMSD of ACh heavy atoms during the M_1_R-ACh binary complex MD simulation and COM distance between the COM of ACh during the MD simulations and that of the initial docking orientation; Figure S6: Two-dimensional plot of COM distance between residues R123 and E360 versus the RMSD of the NPxxY motif with reference to the inactive conformation along the MD simulation for site AS34 and AS56 binding of lithocholic acid and lithocholyltaurine; Figure S7: RMSFs of all residues in M_1_R through MD simulations of M_1_R-ACh and M_1_R-ACh-lithocholic acid or M_1_R-ACh-lithocholyltaurine considering both the AS34 and AS56 sites; Figure S8: LMI residue correlation plots calculated from MD simulations on M_1_R-ACh-lithocholic acid and the M_1_R-ACh-lithocholyltaurine complex targeting both the AS34 and AS56 allosteric sites; Figure S9: RMSD of ACh heavy atoms along the MD simulation for AS34- and AS56-bound lithocholic acid or lithocholyltaurine systems; atomic level of detail observed in the MD trajectory for activation of M_1_R.

## Abbreviations

The following abbreviations are used in this manuscript:

M_1_R	M_1_ muscarinic receptor
ACh	Acetylcholine
POPC	1-palmitoyl-2-oleoyl-sn-glycero-3-phosphocholine
SILCS	Site identification by ligand competitive saturation
MD	Molecular dynamics
FEP	Free energy perturbation
LGFE	Ligand grid free energy
MC	Monte Carlo
PDB	Protein data bank
LMI	Linear mutual information
RMSD	Root-mean-square deviation
RMSF	Root-mean-square fluctuation
COM	Center-of-mass
