# Supplementary material for: Bile Acids Are Potential Negative Allosteric Modulators of M1 Muscarinic Receptors
Source: Biomolecules. 2025 Sep 17;15(9):1326. doi: 10.3390/biom15091326 (PMC12467027; doi:10.3390/biom15091326)
Supplement: Supplementary file 1 [file biomolecules-15-01326-s001.zip › biomolecules-3825636-supplementary.pdf]

Supporting Information

Bile Acids are Potential Negative Allosteric Modulators of M_1 Muscarinic Receptors

Wenbo Yu [1,2,3,*], Alexander D. MacKerell, Jr [1,2,3], David J. Weber [2,3,4,5], and Jean-Pierre Raufman [5,6,7,*]

[1]Computer-Aided Drug Design Center, Department of Pharmaceutical Sciences, School of Pharmacy, University of Maryland Baltimore, Baltimore, MD 21201, USA.

[2]Institute for Bioscience and Biotechnology Research (IBBR), Rockville, MD 20850, USA.

[3]Center for Biomolecular Therapeutics (CBT), School of Medicine, University of Maryland Baltimore, Baltimore, MD 21201, USA.

[4]Department of Biochemistry and Molecular Biology, University of Maryland School of Medicine, Baltimore, MD 21201, USA.

[5]Marlene and Stewart Greenebaum Cancer Center, University of Maryland School of Medicine, Baltimore, MD 21201, USA.

[6]VA Maryland Healthcare System, Baltimore, MD 21201, USA.

[7]Department of Medicine, Division of Gastroenterology and Hepatology, University of Maryland School of Medicine, Baltimore, MD 21201, USA.

*Correspondence: wyu@rx.umaryland.edu (W.Y.); jraufman@som.umaryland.edu (J-P.R.)

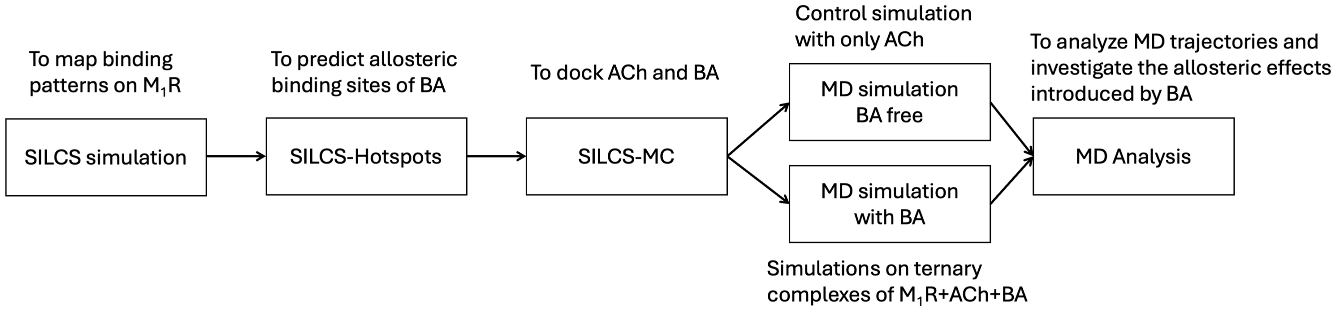

Scheme S1. The computational pipeline performed in the current study to explore potential allosteric interactions of bile acids (BA) with M_1 muscarinic receptors (M_1R). Abbreviations: ACh, acetylcholine; MD, molecular dynamics.

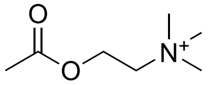

Acetylcholine

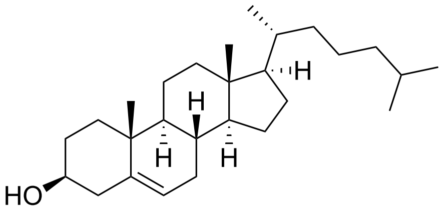

Cholesterol

Primary bile acids

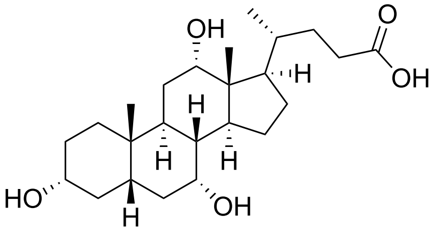

Cholic acid

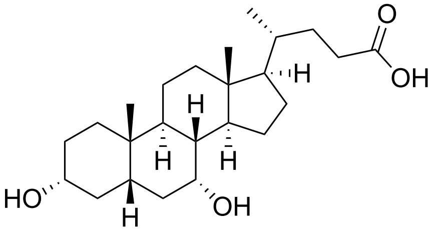

Chenodeoxycholic acid

Secondary bile acids

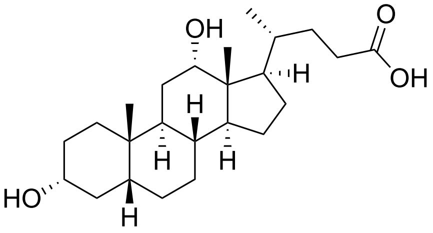

Deoxycholic acid

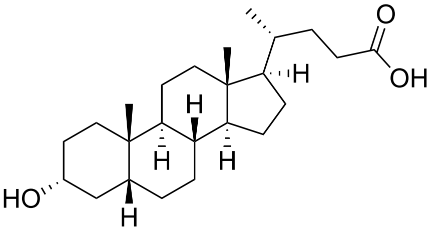

Lithocholic acid

+ 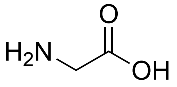

Glycine conjugate

+ 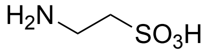

Taurine conjugate

Example:

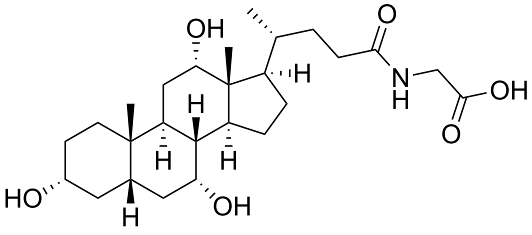

Cholic acid glycine conjugate

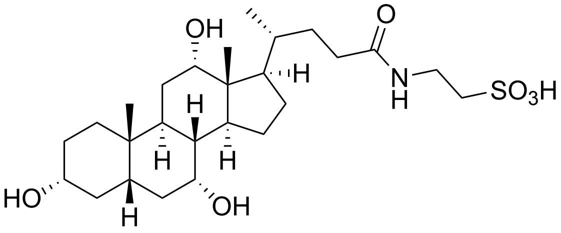

Cholic acid taurine conjugate

Diagram S1. 2D chemical structures of molecules studied in the current work.

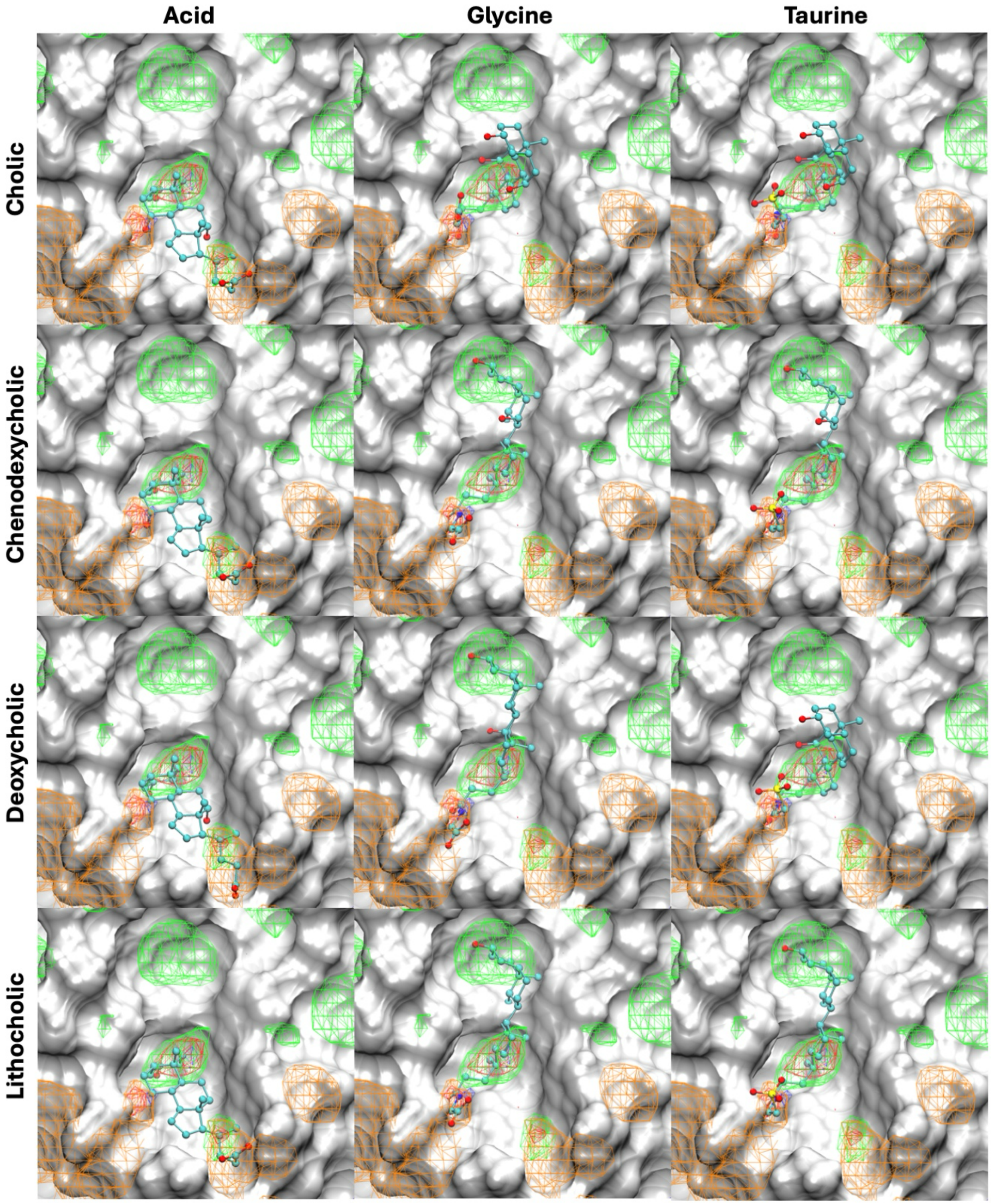

Figure S1. Predicted binding poses for all considered bile acid and glycine and taurine conjugates at the AS34 site using SILCS-MC. SILCS FragMaps are shown with apolar, hydrogen bond acceptor and negatively charged ACEC FragMaps are rendered at a GFE level of -1.0, -1.0 and

-1.2 kcal/mol and colored in green, red, blue, orange, and cyan, respectively. The protein is shown in the surface representation.

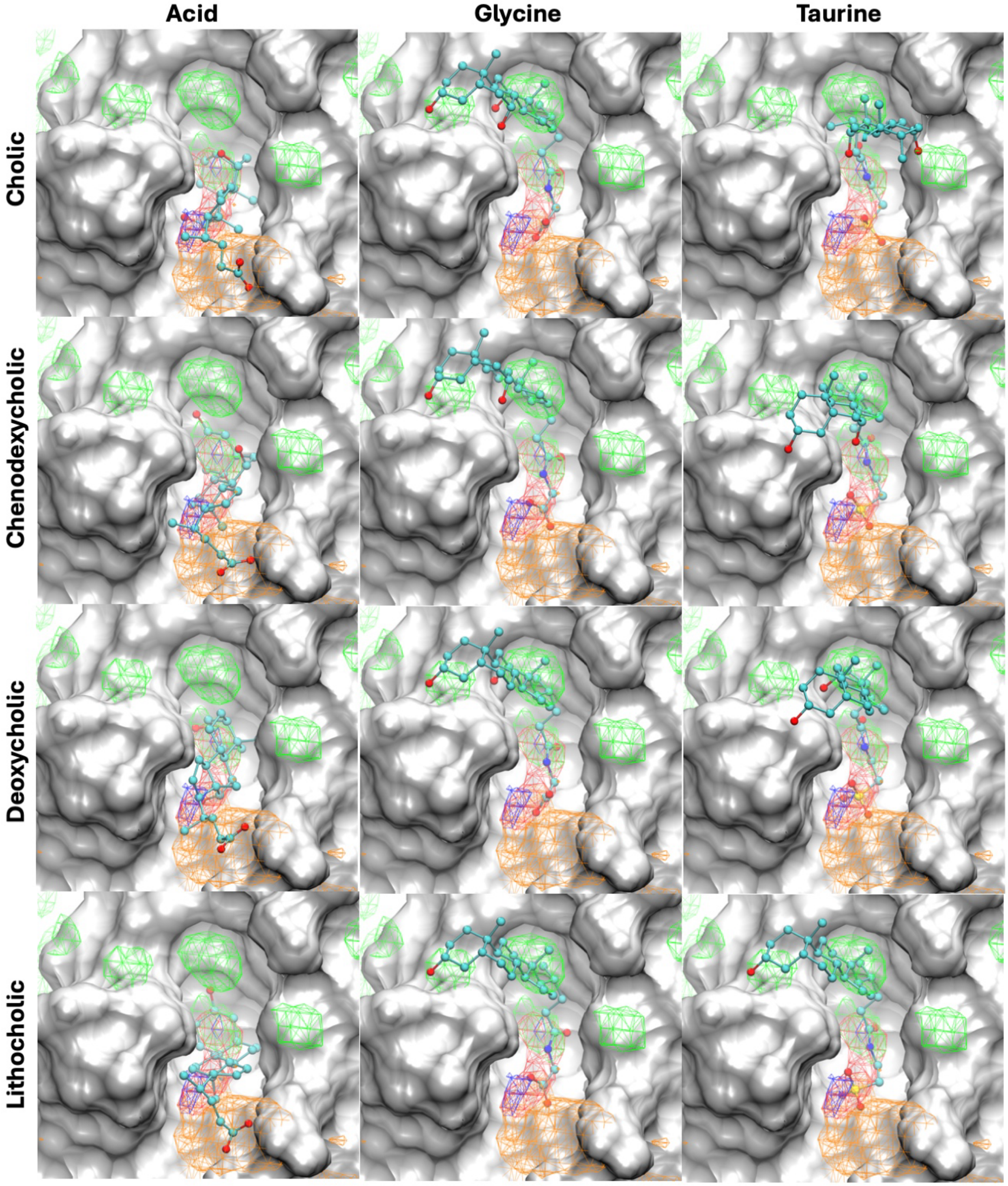

Figure S2. Predicted binding poses for all considered bile acid and glycine and taurine conjugates at the AS56 site using SILCS-MC. SILCS FragMaps are shown with apolar, hydrogen bond

acceptor and donor, and negatively charged ACEC FragMaps are rendered at a GFE level of -1.0, -1.0, -1.0 and -1.2 kcal/mol and colored in green, red, blue, orange, and cyan, respectively. The protein is shown in the surface representation

Table S1. Functional group GFE contributions to LGFE (kcal/mol) for all bile acids and their glycine and taurine conjugates at the two predicted allosteric binding sites on M_1R. 2D structure of lithocholic acid is shown to illustrate defined molecular portions for GFE analysis.

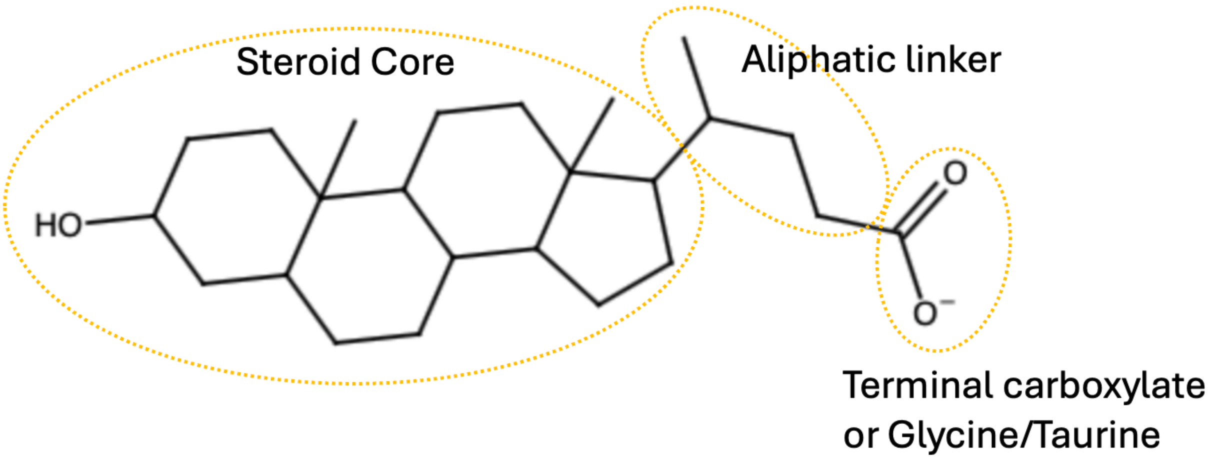

Acid	AS34				AS56			
	Steroid core	Aliphatic linker	Terminal carboxylate	Glycine/ Taurine	Steroid core	Aliphatic linker	Terminal carboxylate	Glycine/ Taurine
Cholic	-6.83	-1.47	-2.00	N/A	-4.61	-0.50	-2.30	N/A
Chenodeoxycholic	-7.34	-1.30	-2.00	N/A	-4.80	-0.60	-2.30	N/A
Deoxycholic	-6.30	-1.30	-1.80	N/A	-5.49	-0.60	-2.10	N/A
Lithocholic	-6.71	-1.20	-2.00	N/A	-4.58	-0.60	-2.30	N/A
Glycine								
Cholic	-5.55	-1.90	N/A	-3.80	-3.61	-0.00	N/A	-5.00
Chenodeoxycholic	-5.84	-2.10	N/A	-4.00	-4.84	-0.10	N/A	-5.00
Deoxycholic	-5.19	-2.00	N/A	-3.80	-4.26	-0.00	N/A	-5.30
Lithocholic	-6.20	-2.10	N/A	-4.00	-5.49	-0.00	N/A	-4.80
Taurine								
Cholic	-5.73	-1.90	N/A	-3.70	-4.05	-0.40	N/A	-5.30
Chenodeoxycholic	-5.63	-2.10	N/A	-3.80	-4.70	-0.10	N/A	-5.50
Deoxycholic	-5.69	-1.90	N/A	-3.70	-4.05	-0.30	N/A	-5.60
Lithocholic	-6.22	-2.10	N/A	-3.80	-5.72	-0.00	N/A	-5.00

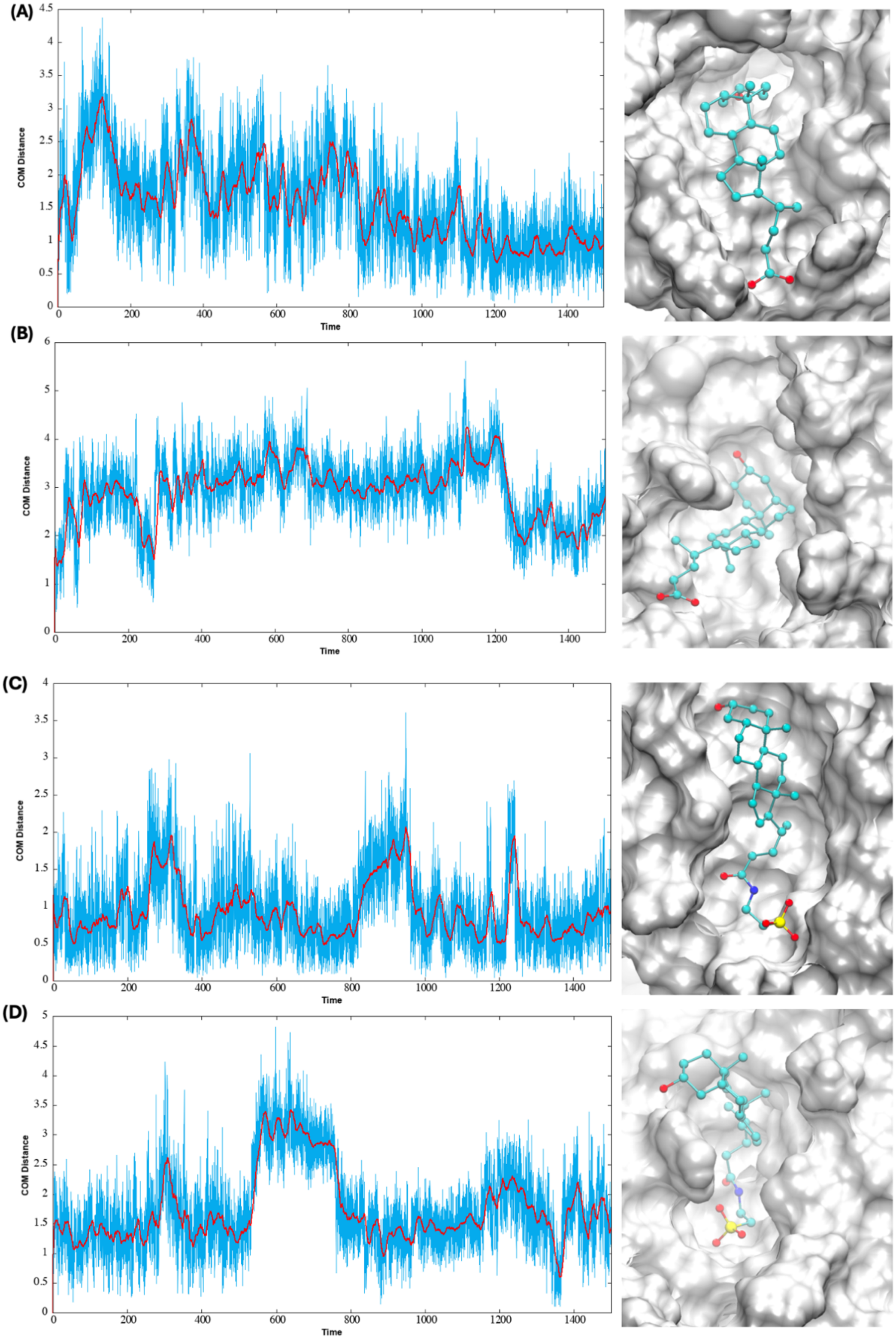

Figure S3. COM distance (in Å) between lithocholic acid (A)&(B) or lithocholyltaurine (C)&(D) during the MD simulations (time in ns) and its initial docking orientation, and the binding pose

from the last frame of MD simulations for site AS34 (A)&(C) and AS56 (B)&(D). Moving average values of distances calculated every 15 ns are also shown as red lines.

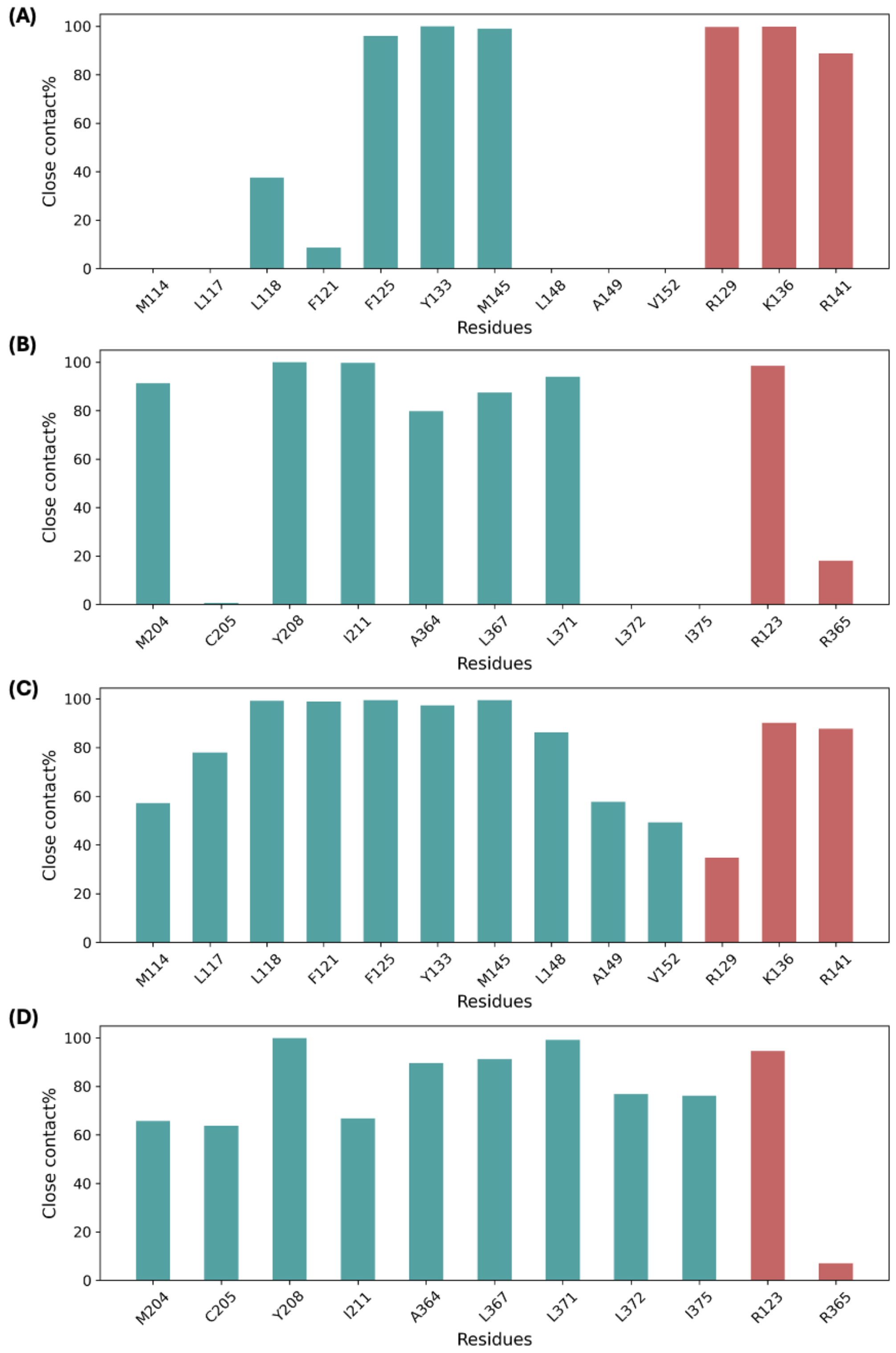

Figure S4. Percentage of close contact formed by lithocholic acid (A) & (B) or lithocholyltaurine (C) & (D) with binding site residues along the whole MD simulation for site AS34 (A) & (C) and AS56 (B) & (D). Close contact is defined as atoms in the ligand found within 3 Å of atoms in the protein residue. Hydrophobic residues are colored green and basic residues red.

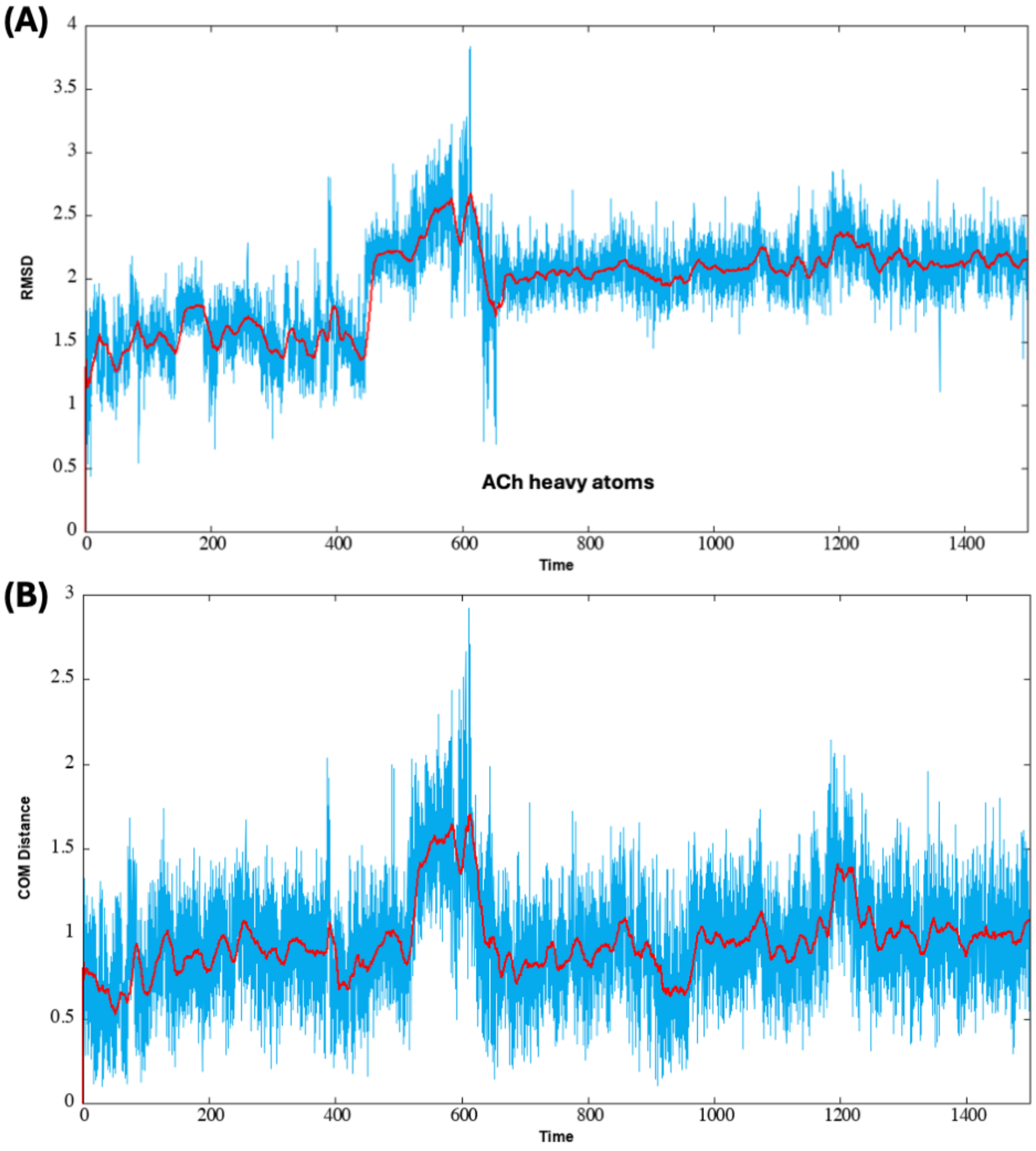

Figure S5. (A) RMSD (in Å) of ACh heavy atoms during the M_1R-ACh binary complex MD simulation with time shown in ns. (B) COM distance (in Å) between ACh during the MD simulations (time in ns) and its initial docking orientation. Moving average values calculated every 15 ns are also shown as red lines. Fluctuations in the RMSD plot for ACh indicates that the ACh ligand undergoes local reorientation while the plateau after about 620 ns indicates that the binding of ACh assumes a stable orientation in the orthosteric binding site in the later stage of MD simulation.

The overall small RMSD and COM distance values indicate that its binding orientation does not deviate too much from our docking pose as predicted using SILCS-MC.

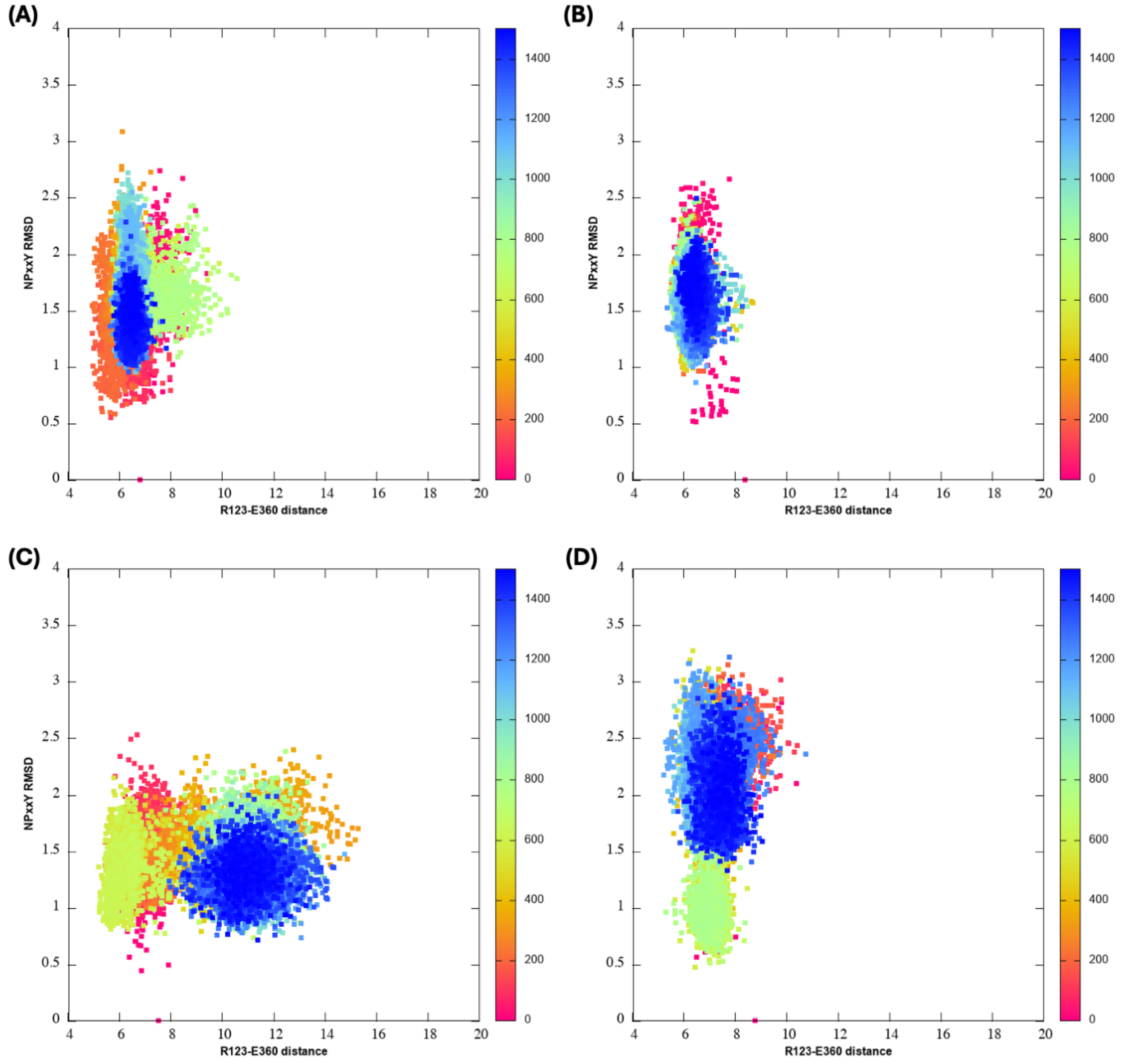

Figure S6. Two-dimensional plot for COM distance (in Å) between residue R123 and E360 versus RMSD (in Å) of the NPxxY motif with reference to the inactive conformation along the MD simulation for site AS34 (A) & (C) and AS56 (B) & (D) binding of lithocholic acid (A) & (B) and lithocholyltaurine (C) & (D).

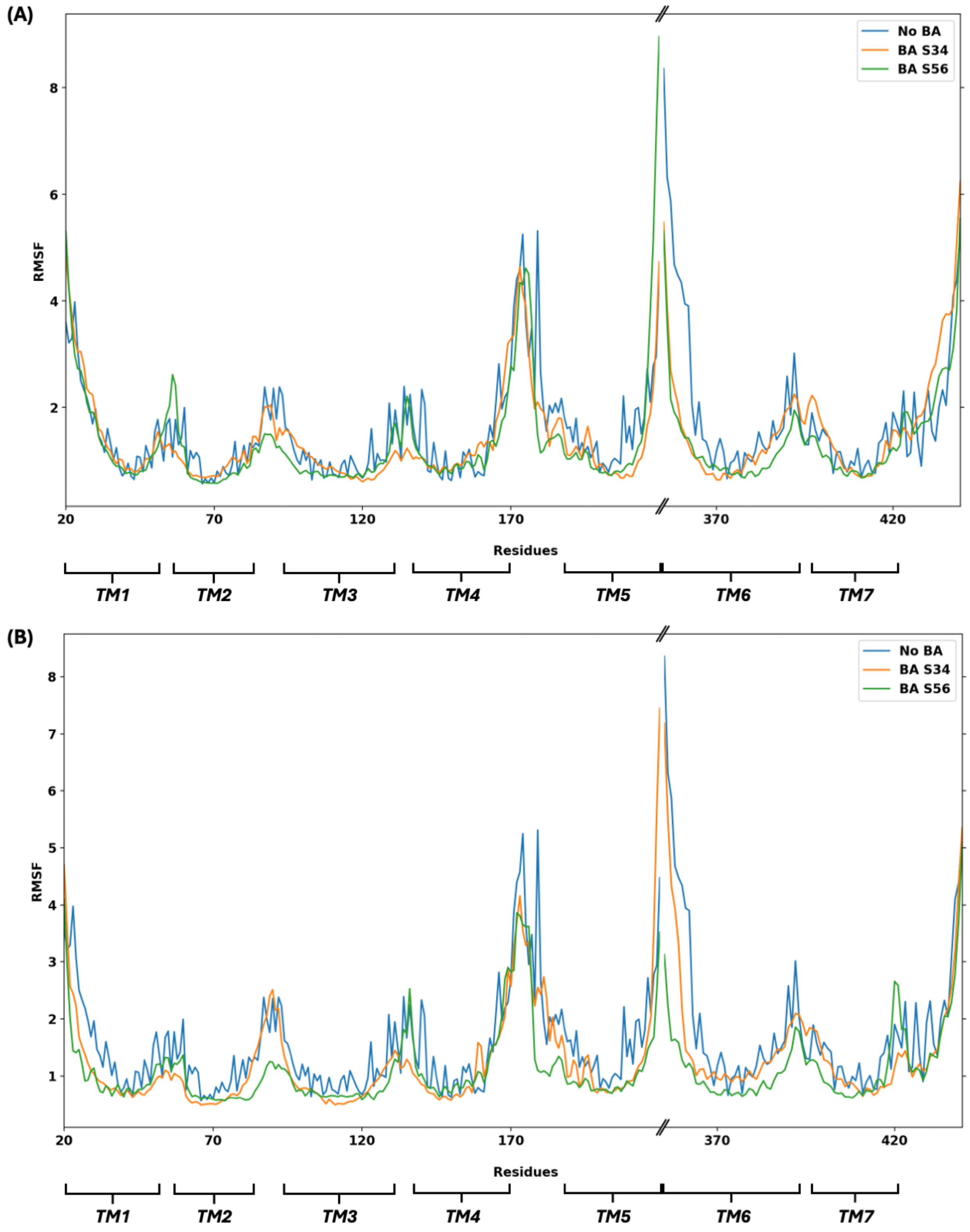

Figure S7. Root-mean-square fluctuations (RMSF) of all residues in M_1R through MD simulations of M_1R-ACh and M_1R-ACh-lithocholic acid (A) or M_1R-ACh-lithocholyltaurine (B) considering both AS34 and AS56 sites.

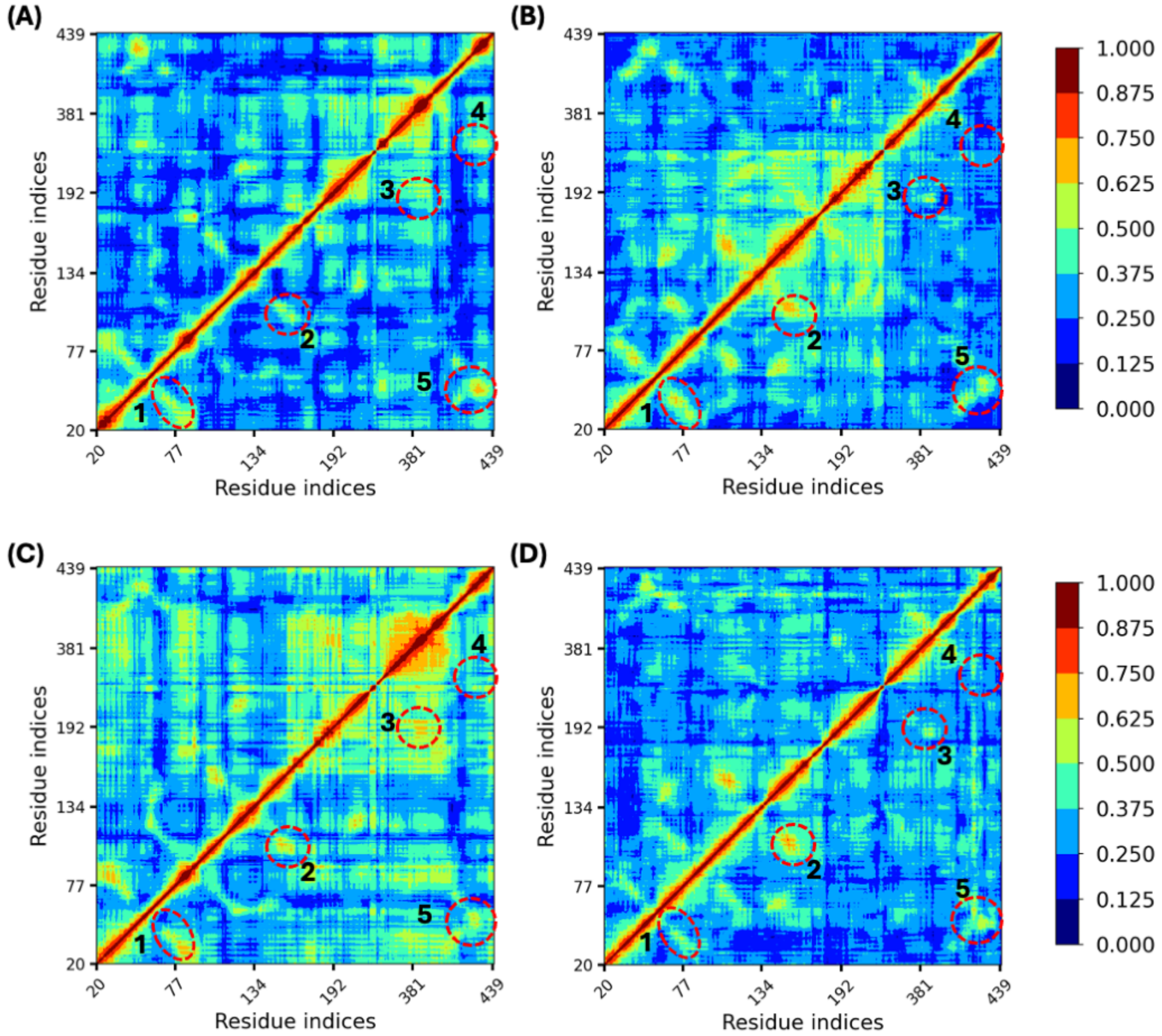

Figure S8. Linear mutual information (LMI) residue correlation plots calculated from MD simulations on M_1R-ACh-lithocholic acid (A) & (B) and M_1R-ACh-lithocholyltaurine (C) & (D) complex targeting both AS34 (A) & (C) and AS56 (B) & (D) allosteric site. Five off-diagonal regions with high LMI values identified for M_1R-ACh complex simulation are indicated by red dashed circles.

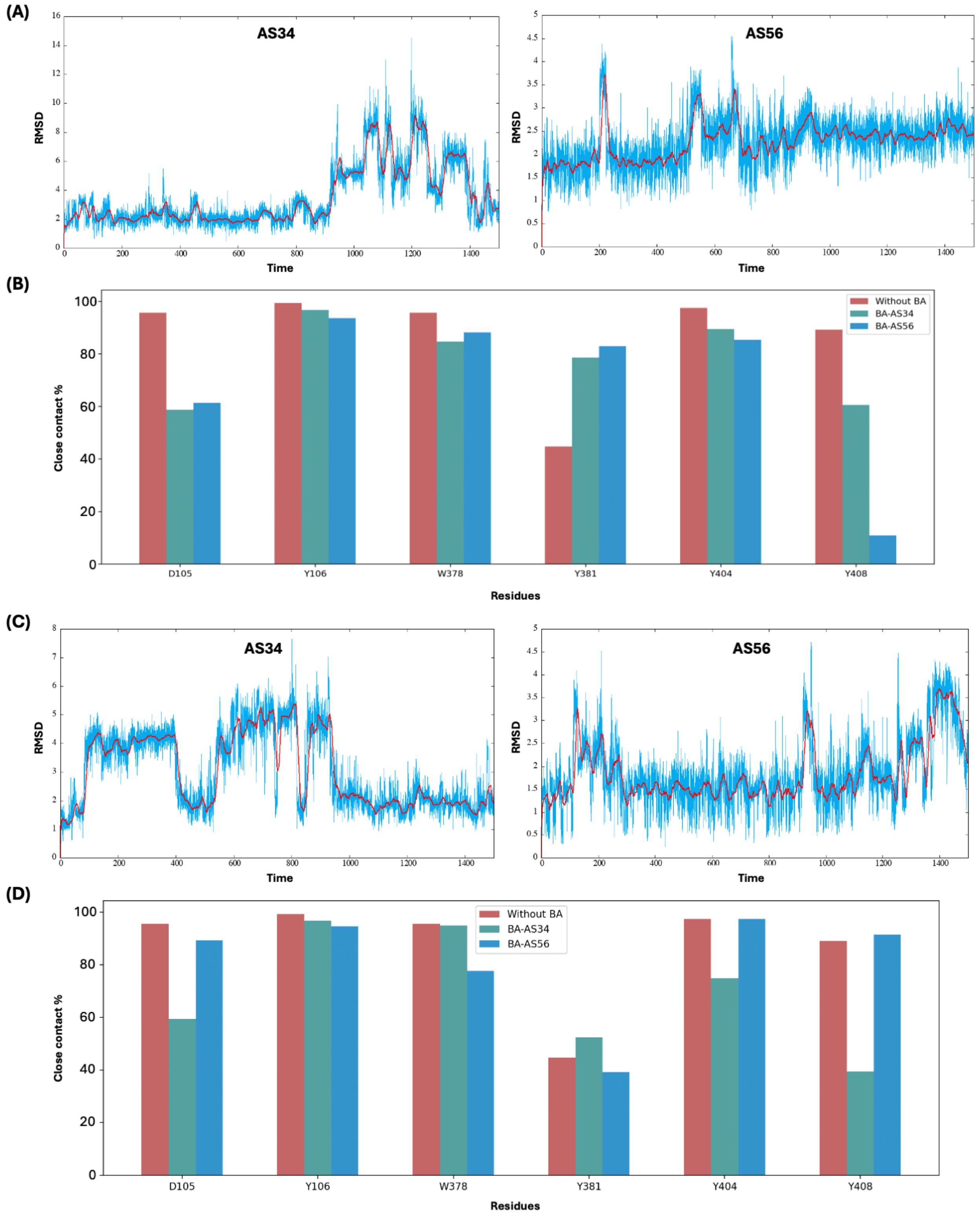

Figure S9. RMSD (in Å) of ACh heavy atoms along the MD simulation (in ns) for AS34 and AS56 bound lithocholic acid (A) or lithocholyltaurine (C) systems. Moving average values of distances calculated every 15 ns are also shown as red lines. Percentage of close contact formed by ACh

with active site residues along the whole MD simulation for M_1R-ACh and M_1R-ACh-lithocholic acid (B) or lithocholylglycine (D) systems for both AS34 and AS56 sites.

Atomic level of details observed in the MD trajectory for activation of M_1R

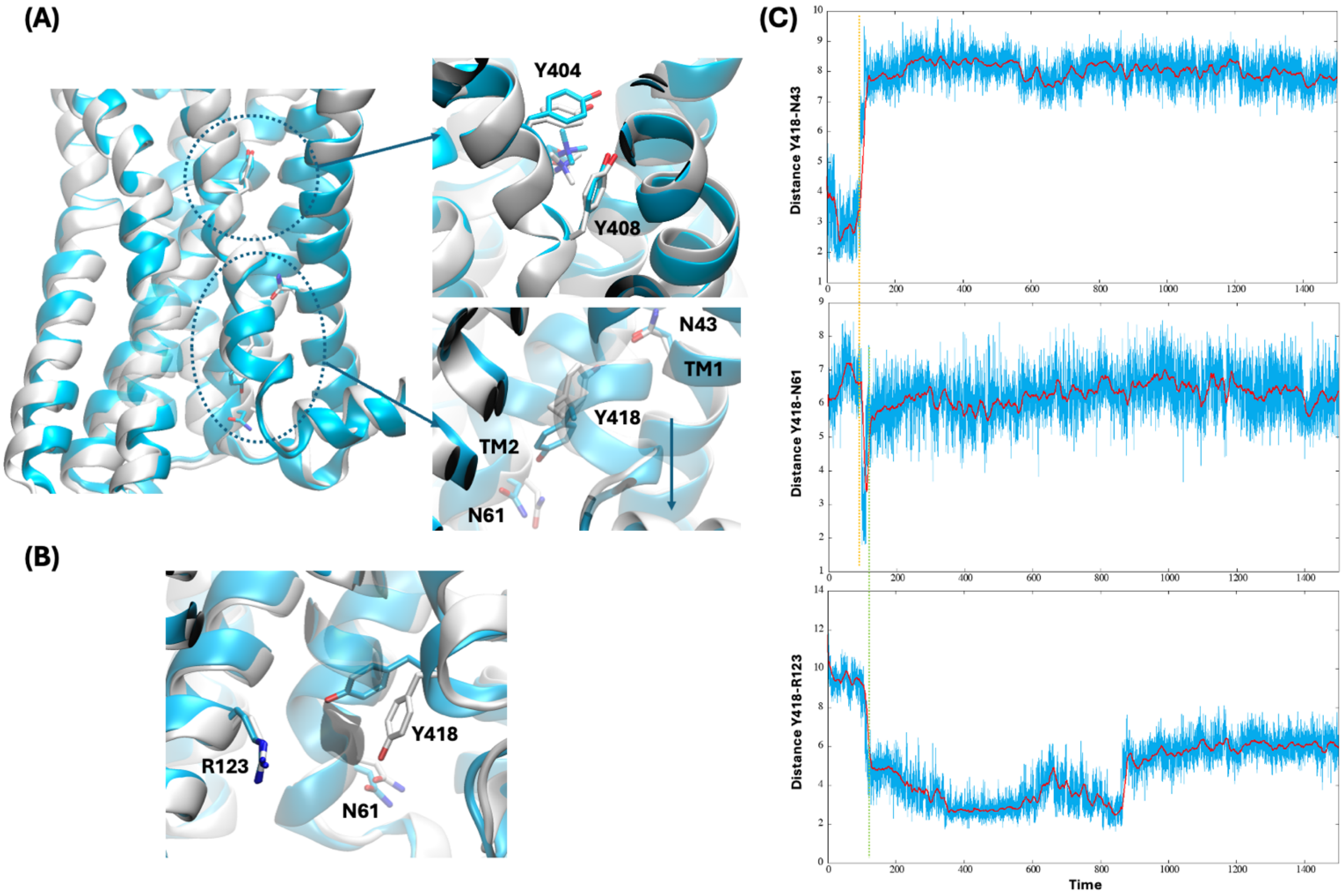

Figure SS1. (**A**) Comparison of two neighboring MD frames at 97 ns separated by 2 ps. The first frame is shown in white, and the second frame is in cyan. Close up views on the right illustrate the extracellular (top) and intracellular (bottom) regions of the protein. The arrow indicates a downward movement of the intracellular region of TM7. Some regions of the protein are rendered in transparent, so key residues can be observed. (**B**) Comparison of two neighboring MD frames at 114 ns. (**C**) COM distances (in Å) between Y418 and other residues along the MD simulation (time in ns). Orange vertical dashed line indicates the time when Y418-N43 hydrogen bonding was lost, and Y418-N61 hydrogen bonding interaction was formed. Green vertical dashed line indicates the time when the Y418-N61 interaction was broken while Y418-R123 interaction was formed. Moving average values of distances calculated every 15 ns are also shown as red lines.

The first large conformational change observed for residue Y418 in the NPxxY motif on the intracellular side of TM7 happened as early as 97 ns in the MD simulation. As shown in Figure SS1(A), the binding of ACh caused distortions in TM7 and pushed its intracellular component downward. This change breaks the hydrogen bond formed between Y418 with N43 on TM1 in the inactive structure and Y418 forms a new hydrogen bond interaction between N61 on TM3. However, different from Miao's study (Miao et al. 2013) on the apo structure of M_2R protein, large conformational changes of the active site residue Y408 was not observed since Y408 was stabilized by an interaction network that formed upon binding of the agonist. This suggested that large distortion of active site residue Y408 is not necessary to cause conformational changes in the intracellular half of TM7 of M_1R. For the apo form of MR protein as studied by Miao et. al. (Miao et al. 2013), a large conformational change of Y430 of M_2R (corresponds to Y408 in M_1R) was observed due to the absence of ligand at the active site. Upon binding of the agonist ACh to M_1R, Y408 remains in a position similar to that when the antagonist is present, indicating that both agonist and antagonist are accommodated well in the tyrosine cage.

Later in the MD trajectory, as shown in Figure SS1(B) and SS1(C), Y418 did not interact for long with N61 and at around 114 ns, with further distortion in the NPxxY motif, Y418 was pushed toward the ionic residue R123 and formed a new hydrogen bond. Y418 remained in this position until the end of 1.5 μs MD simulation (Figure SS1(C)).

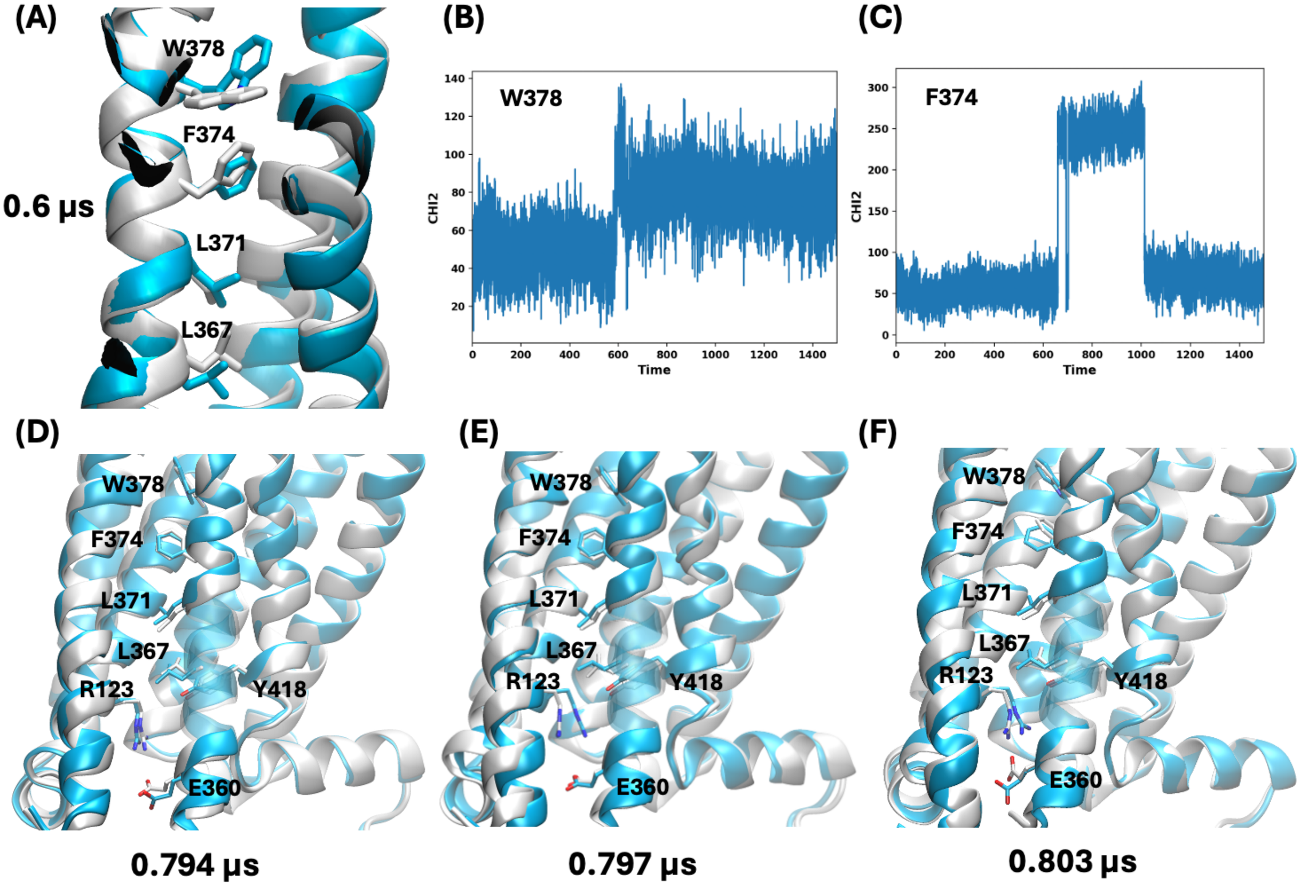

Figure SS2. (**A**) Two neighboring MD frames at 600 ns from the trajectory. The older frame is shown in white, and the newer frame is shown in cyan. Key residues on TM6 are labeled. Chi2 angle of W378 (**B**) and F374 (**C**) along the MD simulation are plotted. MD frames at 794 ns (**D**), 797 ns (**E**) and 803 ns (**F**) are also shown. A portion of the protein is rendered in transparent to allow key residues to be observed.

Moving ahead in the MD simulation, the next important conformational change event observed is the break of the ionic lock. This happened sequentially after the distortion of NPxxY motif as suggested by Figure 5 (C) and as described in the main text. As addressed above, the conserved residue W400 in M_2R (corresponds to W378 in M_1R) was identified as a key residue in the conformational dynamics of TM6 during activation. Gong et. al. proposed a pipeline on how residues on TM6 transduce conformational dynamics across the membrane to the cytoplasmic side of the protein (Gong et al. 2024). They suggested the interaction between W400 and F396 in M_2R and their coupled structural changes can cause a sequential induced conformational change across TM6. Specifically, the aromatic ring of W400 stays in a vertical orientation to allow aromatic ring flipping motions of F396 to induce conformational changes on other hydrophobic residues such as I389 and L393 down the TM6 helix. These motions are essential for the outward movement of TM6. Such an observation is confirmed by our simulation for M_1R. As shown in Figure SS2 (A) to (C), at ~600 ns, residue

W378 deviated from a horizontal orientation of its aromatic ring with Chi2 angle ~50 degrees found in the inactive state conformation. It then began to adopt a vertical orientation with Chi2 angle remaining ~90 degrees until the end of MD simulation. Shortly after this event, at ~650 ns, the aromatic ring of residue F374 flipped with its Chi2 angle changing from ~50 to ~250 degrees. It flipped over again at ~1 μs. Such a conformational change induced coupling between W378 and F374 that induce conformational distortions to other residues such as L371 and L367 and further affect stability of ionic lock residue E360 down the TM6 helix. However, in our trajectory, there is another contributing factor not mentioned in previous studies, which help break the ionic lock. This refers to a hydrogen bond that occurs between the ionic lock residue R123 with Y418, which also affects the stability of the ionic lock. The two contributors each target and distort one of the residues comprising the ionic lock, resulting in its destabilization.

Both mechanisms were observed frequently in the trajectory from ~600 to ~960 ns as indicated by the widely distributed ionic lock residue distances shown in Figure 5(C) in the main text. For example, Figure SS2(D) shows two neighboring frames where large structural distortions are seen for L371 and L367 and this further affects E360 where it was pushed downward away from R123 while R123 was not distorted by Y418 yet. At ~797 ns, as shown in Figure SS2(E), R123 was distorted by interaction with Y418 while E360 was not affected by conformational dynamics on TM6. And later, at ~803 ns, as shown in Figure SS2(F), both ionic lock residues R123 and E360 were affected by TM6 dynamics and Y418 involved hydrogen bond. At ~940 ns, the ionic lock was fully broken, and the two residues remained separated until the end of the simulation.

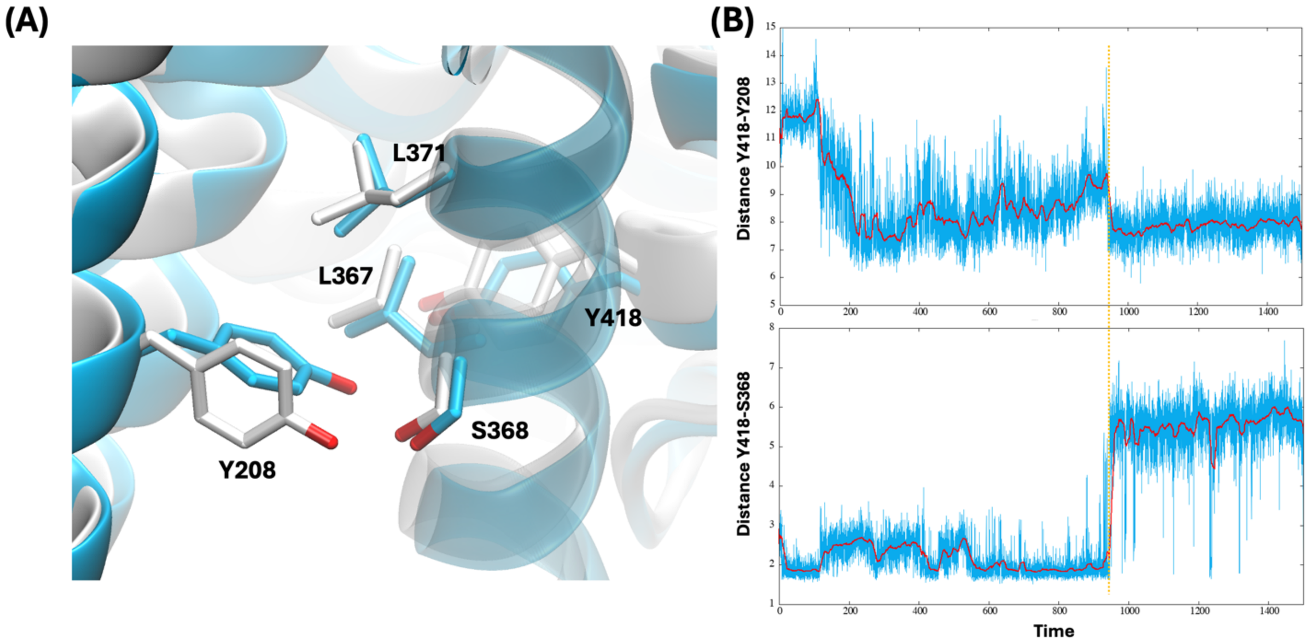

Figure SS3. (**A**) Two neighboring MD frames at 942 ns from the trajectory. Old frame is shown in white and new frame shown in cyan. A portion of the protein is shown in

transparent representation to show key residues. (**B**) COM distances between Y418 and Y208 as well as between Y418 and S368 along the MD simulation. Moving average values of distances, calculated every 15 ns, are shown as red lines. Orange vertical dashed line indicate the time when Y208 reoriented into the gap between TM5 and TM6 and moved closer toward Y418.

Upon break of the ionic lock, the coupling between TM6 and TM3 was weakened, and this opened space between TM5 and TM6, which allows for more conformational dynamics involving residue Y208. In the inactive state, residue Y208 on TM5 has its sidechain facing the lipid environment and forms a hydrogen bond between its sidechain hydroxyl group and residue S368 on TM6. At ~942 ns in the trajectory, break of this interaction was observed as shown in Figure SS3 with the Y208 sidechain shifted into the gap between TM5 and TM6 and got closer to residue Y418. Note that Y208 forms hydrogen bond with Y418 in the crystal structure of active state M_1R.

In summary, according to our MD simulation, M_1R activation upon binding of the agonist ACh consists of three important sequential steps. First, distortion of the NPxxY motif causes Y418 to appear in proximity of R123. Next, conformational dynamics of resides W378, F374, L371 and L367 on TM6 distort E360, together with interaction between Y418 and R123 weaken and eventually cause the ionic lock to break. Following the break of the ionic lock, increased flexibility of Y208 enables its reorientation to approximate Y418 and form hydrogen bonding interaction in the active state.

Reference:

Miao Y, Nichols SE, Gasper PM, Metzger VT, McCammon JA. Activation and dynamic network of the M2 muscarinic receptor. Proceedings of the National Academy of Sciences. 2013 Jul 2;110(27):10982–7.

Gong Z, Zhang X, Liu M, Jin C, Hu Y. Visualizing the M2 muscarinic acetylcholine receptor activation regulated by aromatic ring dynamics. bioRxiv. 2024 Jan 1;2024.02.18.580854
